# Systemic pharmacological treatment of digital ulcers in systemic sclerosis: a systematic literature review

**DOI:** 10.1093/rheumatology/kead289

**Published:** 2023-06-19

**Authors:** Laura Ross, Nancy Maltez, Michael Hughes, Jan W Schoones, Murray Baron, Lorinda Chung, Dilia Giuggioli, Pia Moinzadeh, Yossra A Suliman, Corrado Campochiaro, Yannick Allanore, Christopher P Denton, Oliver Distler, Tracy Frech, Daniel E Furst, Dinesh Khanna, Thomas Krieg, Masataka Kuwana, Marco Matucci-Cerinic, Janet Pope, Alessia Alunno

**Affiliations:** Department of Medicine and Rheumatology, University of Melbourne at St Vincent’s Hospital, Melbourne, Victoria, Australia; Department of Medicine, Division of Rheumatology, Ottawa Hospital, University of Ottawa, Ottawa, Ontario, Canada; Department of Rheumatology, Northern Care Alliance NHS Foundation Trust, Salford Care Organisation, Salford, UK; Division of Musculoskeletal and Dermatological Sciences, University of Manchester, Manchester, UK; Directorate of Research Policy (formerly Walaeus Library), Leiden University Medical Center, Leiden, The Netherlands; Division of Rheumatology, Jewish General Hospital, McGill University, Montreal, Quebec, Canada; Department of Rheumatology, Stanford University School of Medicine and Palo Alto VA Health Care System, Palo Alto, CA, USA; Department of Rheumatology, Division of Rheumatology, University of Modena and Reggio Emilia, Policlinico of Modena, Modena, Italy; Department of Dermatology and Venereology, University Hospital Cologne, Cologne, Germany; Department of Rheumatology and Rehabilitation, Assiut University Hospital, Assiut, Egypt; Department of Immunology, Rheumatology, Allergy and Rare Diseases, Raffaele Hospital, Vita-Salute San Raffaele Università, Milan, Italy; Department of Rheumatology, Cochin Hospital, AP-HP, Paris Descartes University, Paris, France; Centre for Rheumatology and Connective Tissue Diseases, Royal Free Hospital, London, UK; UCL Division of Medicine, University College London, London, UK; Department of Rheumatology, University Hospital Zurich, University of Zurich, Zurich, Switzerland; Department of Medicine, Division of Rheumatology and Immunology, Vanderbilt University Medical Center, Veterans Affairs Medical Center, Nashville, TN, USA; Department of Rheumatology and Medicine, University of California, Los Angeles, Los Angeles, CA, USA; Department of Rheumatology, University of Michigan, Ann Arbor, MI, USA; Department of Rheumatology, Division of Rheumatology, University of Modena and Reggio Emilia, Policlinico of Modena, Modena, Italy; Department of Allergy and Rheumatology, Nippon Medical School, Tokyo, Japan; Department of Experimental and Clinical Medicine, Division of Rheumatology, Careggi University Hospital, University of Florence, Florence, Italy; Schulich School of Medicine and Dentistry, University of Western Ontario, London, Ontario, Canada; Department of Life, Health and Environmental Sciences, University of L’Aquila, L’Aquila, Italy; Internal Medicine and Nephrology Division, ASL1 Avezzano-Sulmona-L'Aquila, L'Aquila, Italy

**Keywords:** SSc (scleroderma), digital ulcers, management, pharmacotherapy, systemic treatment

## Abstract

**Objective:**

To evaluate the evidence concerning systemic pharmacological treatments for SSc digital ulcers (DUs) to inform the development of evidence-based treatment guidelines.

**Methods:**

A systematic literature review of seven databases was performed to identify all original research studies of adult patients with SSc DUs. Randomized controlled trials (RCTs) and prospective longitudinal observational studies (OBSs) were eligible for inclusion. Data were extracted, applying the patient, intervention, comparison, outcome framework, and risk of bias (RoB) was assessed. Due to study heterogeneity, narrative summaries were used to present data.

**Results:**

Forty-seven studies that evaluated the treatment efficacy or safety of pharmacological therapies were identified among 4250 references. Data from 18 RCTs of 1927 patients and 29 OBSs of 661 patients, at various RoB (total 2588 patients) showed that i.v. iloprost, phosphodiesterase-5 inhibitors and atorvastatin are effective for the treatment of active DUs. Bosentan reduced the rate of future DUs in two RCTs (moderate RoB) and eight OBSs at low to high RoB. Two small studies (moderate RoB) indicate that Janus kinase inhibitors may be effective for the treatment of active DUs, otherwise there are no data to support the use of immunosuppression or anti-platelet agents in the management of DUs.

**Conclusion:**

There are several systemic treatments, across four medication classes, that are effective therapies for the management of SSc DUs. However, a lack of robust data means it is not possible to define the optimal treatment regimen for SSc DUs. The relatively low quality of evidence available has highlighted further areas of research need.

Rheumatology key messages

- Therapeutic strategies for the management of SSc DUs vary significantly.
- Bosentan can prevent future DUs and iloprost, phosphodiesterase-5 inhibitors and atorvastatin can treat active DUs.
- Limited high-quality data exist to support DU management guidelines, highlighting areas of future research need.

## Introduction

Digital ulcers (DUs) affect approximately half of patients with SSc, resulting in significant pain and disability [1, 2]. DUs, particularly those that develop on the fingertips, are believed to be the result of peripheral microvascular ischaemia [3]. There is now a wide range of drug therapies available for the prevention and treatment of new DUs. A primary aim of the World Scleroderma Foundation (WSF) Digital Ulcer Working Group is to develop evidence-based treatment recommendations to optimize DU management in clinical practice. In this context, we present the findings of a systematic literature review (SLR) to investigate the efficacy and safety data of systemic pharmacological therapies for SSc DUs.

## Methods

This SLR was performed in accordance with the Preferred Reporting Items for Systematic Reviews and Meta-Analysis (PRISMA) checklist [4]. A systematic literature search of PubMed, MEDLINE (OVID), Embase (OVID), Web of Science, Cochrane Library, Emcare (OVID) and Academic Search Premier databases from inception to 26 August 2022 was performed. Data from unpublished studies or conference abstracts and proceedings were not included in this review. We sought to identify original research studies of adult populations with SSc DUs treated with systemic pharmacological treatment. A patient, intervention, comparison, outcome (PICO) model was applied to the research questions and search strategy, which are detailed in Supplementary Data S1 and S2, available at *Rheumatology* online.

Based on the PICO framework, studies were eligible for inclusion if they included adult (age ≥18 years) patients with definite SSc and reported DU outcomes as either a primary or secondary endpoint. Prospective studies, of any design, were included. Outcomes of interest were the treatment of active DUs, including number of DUs and healing rates of DUs, as well as prevention of new DUs and treatment safety data. Only manuscripts published in English were included in the final review.

All abstracts were independently screened by two reviewers (L.R. and N.M.). The full text of all eligible citations was then independently assessed by the same reviewers (see Supplementary Data S3 for all full text articles excluded) and relevant study data extracted according to a prespecified template (Supplementary Data S4, both available at *Rheumatology* online). Any disagreement between reviewers was resolved by consensus. Due to the extensive interstudy heterogeneity in relation to both methodology and outcome measurement, meta-analysis of results was not possible and narrative summaries were used to present the data. The risk of bias (RoB) of randomized controlled trials (RCTs) was assessed using the Cochrane RoB tool [5] and the RoB in Non-Randomized Studies of Interventions (ROBINS-I) tool [6] was applied to observational studies (OBSs). RoB assessment was performed independently by two authors (L.R. and N.M.). All disagreements were resolved by consensus.

## Results

**Figure 1.**
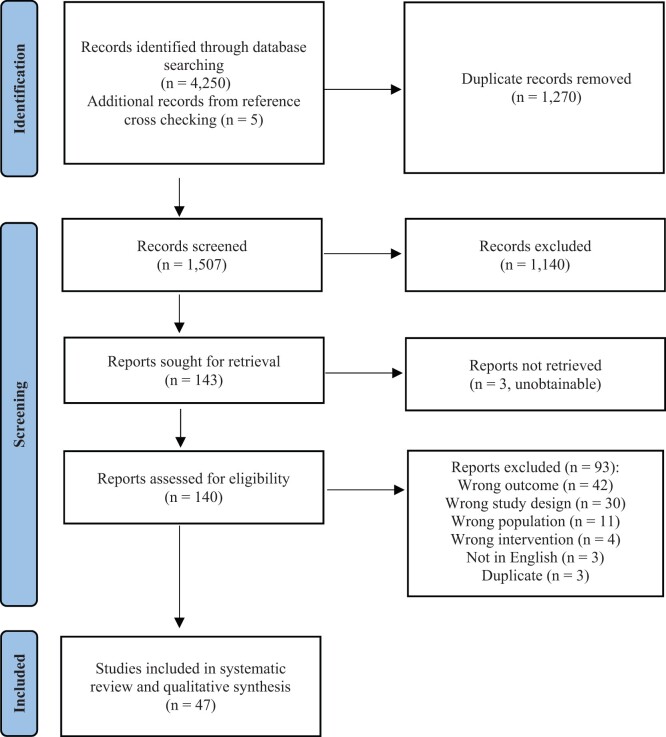
PRISMA flowchart of study selection

The literature search identified 4250 records. After deduplication, 1507 titles and abstracts were screened and 47 articles were eligible for inclusion (Fig. 1). Of these, 18 were RCTs and 29 were prospective OBSs (8 cohort studies, 21 prospective case series). There was a total of 2588 patients studied, of whom 1539 (60.16%) had a DU at the baseline study visit. In 16 (34%) studies, DU was the secondary endpoint (Table 1). Ten (21%) studies did not explicitly state the primary endpoint of interest. A range of pharmacotherapies were evaluated in a various number of studies; with endothelin receptor antagonists (ERAs; *n* = 13), phosphodiesterase-5 inhibitors (PDE5is; *n* = 6), prostacyclin analogues (*n* = 12), calcium channel blockers (CCBs; *n* = 2), atorvastatin (*n* = 2), antiplatelet agents (*n* = 2), immunosuppressive or immunomodulatory agents (*n* = 5) and other (*n* = 4). One study evaluated i.v. iloprost in combination with bosentan. One study compared the use of i.v. prostacyclin analogues and local therapy with botulinum toxin A and compared the costs for each therapy. There was no formal health economic analysis of any therapy for DUs presented in any study. An overview of the main study characteristics is shown in Table 1, with additional details of RCTs provided in Table 2. Additional details of OBSs and RoB assessments of all studies are shown in Supplementary Tables S1 and S2, available at *Rheumatology* online.

**Table 1.** Summary of characteristics of all included studies.

Drug, median (range), year of publication	RCTs				OBSs			
	Patients with DUs at baseline, *n*	Studies with DU primary endpoint, *n* (%)	Primary endpoint	Follow-up, months, median (range)	Patients with DUs at baseline, *n*	Studies with DU primary endpoint, *n* (%)	Primary endpoint	Follow-up, months, median (range)
CCBs [7, 8] RCT: 1987 OBS: 1983	9	1/1 (100)	NR [8]	4.6	4	1/1 (100)	Daily RP frequency and number of DUs [7]	1.4
PDE5i [9–14] RCT: 2016 (2010–2017 OBS: 2011 (2010–2013)	94	1/3 (33)	Change in finger blood flow [11] RCS [9] Time to healing [10]	2.3 (1.4–2.8)	38	2/3 (66)	DU healing [12] NR [14] Safety data [13]	5.2 (3–16.2)
ERA^a^ [15–27] RCT: 2011 (2004–2016) OBS: 2013 (2008–2017)	819	3/3 (100)	Number of new DUs [18–20] Time to healing [18]	3.7 (3.7–7.4)	242	8/10 (80)	Change in mRSS [16] No active or new DUs [21] NR [16, 22, 23] Number of DUs [25, 26] Number of healed DUs [24, 27] Safety data [17] Size of DU [26] Time to healing [15]	12 (5.5–36)
Prostacyclin analogues [28–39] RCT: 2003 (1992–2017) OBS: 2009 (1987–2022)	280^b^	3/6 (50)	Change in net DU burden [36] Daily RP diary [29] DU healing [39] Patients with new DUs [37] RP burden [31] Weekly RP attacks [38]	3.7 (2.1–12)	96^b^	3/6 (50)	Change in DU burden [35] Number of DUs [34] NR [30, 32, 33] Peripheral blood flow [28]	4.15 (0–36.5)
Antiplatelet agents [40, 41] RCT: 1984 OBS: 2016	10	0/1 (0)	Change in general SSc [40]	12	0	0/1 (0)	Suppression of ADP-dependent platelet activation [41]	12
Atorvastatin [42, 43] RCT: 2008 OBS: 2009	45	0/1 (0)	Vascular function [42]	4	3	0/1 (0)	Effects on RP [43]	24
Immunomodulatory agents^c^ [44–50] RCT: 2018 (2016–2021) OBS: 2005	26	0/2 (0)	Number of DU patients^d^ [45] Change in mRSS [44]	12	80	1/5 (20)	NR [46] Change in mRSS [50] Number of DUs [47, 48] Weekly RP attacks [49]	14.8 (1–42)
Riociguat [51] RCT: 2019 OBS: NP	17	1/1 (100)	Change in net ulcer burden [51]	7.4	–	–		–
Ketanserin [52] RCT: NP OBS: 1989	–	–		–	6	0/1 (0)	Pharmacokinetics [52]	6
Combination therapy [53] RCT: NP OBS: 2016	–	–		–	10	0/1 (0)	Fewer new DU [53]	48

a DUAL 1 and 2 studies considered one study as results published in a single publication.

b One study [Vayssairat (RCT), *n* = 107; Rademaker (observational), *n* = 13] did not state the number of patients with DUs at baseline.

c Agents studied: baricitinib, cyclophosphamide, methotrexate, N-acetylcysteine, plasmapheresis and tofacitinib.

d Post hoc analysis of Scleroderma Lung Study data set.

ADP: adenosine diphosphate; NP: not published; NR: not reported.

**Table 2.** Characteristics of included RCTs of systemic pharmacological treatment

Author [ref], year	Participants (*n*); baseline characteristics	Intervention	Study design	Inclusion criteria	Definition of DU	Definition of ulcer healing	Concomitant therapies	Primary outcome	Results
CCBs									
Meyrick Thomas [8], 1987	9	Nifedipine: 10 mg TDS; 6 weeks therapy, 20 weeks observation	Double-blind, placebo-controlled, crossover study	SSc and RP	Not stated	Not stated	None	Not stated	33% of patients developed new DUs on treatment, 66% of patients developed new DUs on treatment (NS)
PDE5i									
Hachulla [10], 2016	83; dcSSc: 47.0%, mean age 49.3 years, mean disease duration 6.9 years	Sildenafil: 20 mg TDS for 12 weeks	Double-blind, placebo-controlled RCT	At least one ischaemic DU present on fingers distal to PIP	Break in skin with loss of epithelialization of distal finger surface of ischaemic origin not located over calcifications or extensor surfaces	Complete re-epithelialization	CCB (61.5%) Bosentan (33.7%)	Time to healing and healing rate	HR for healing 1.33 (*P* = 0.18), adjusted for number of DUs at entry [HR 1.27 (*P* = 0.27)] Fewer number of DUs at weeks 8 and 12 with treatment
Andrigueti [11], 2017	41 (4 patients with active DUs at baseline); dcSSc: 34.15%, mean age 44.69 years, mean disease duration 3.2 years	Sildenafil: 100 mg/day; 8 weeks of treatment and 2 weeks post-treatment follow-up	Double-blind, placebo-controlled RCT	≥1 RP attack/day during week before recruitment despite RP treatment	Not stated	Not stated	CCB (70.73%) ACEI/ARB (34.15%) Aspirin (12.20%) Corticosteroids (36.59%) Cyclophosphamide (14.63%)	Changes in finger blood flow	75% of patients with baseline DUs randomized to treatment; all patients had complete healing of DUs after 8 weeks with no new DUs recorded
Shenoy [9], 2010^a^	24; dcSSc: 75%, mean age 36.87 years, mean disease duration 6.8 years	Tadalafil: 20 mg alternate days for 6 weeks	Double-blind, placebo-controlled, crossover study	≥4 RP attacks per week in 2 weeks preceding recruitment despite vasodilator therapy	Breach in surface epithelium at finger or toe pulps at or distal to PIP joints	Not stated	CCB (100%) ACEI (70.8%) Aspirin (8.3%) Pentoxifylline (4.2%) Corticosteroids (29.2%) Immunosuppression (25%)	Raynaud’s condition score	One new DU during treatment *vs* 13 new DUs during placebo treatment (*P* < 0.001)
ERAs									
Khanna [20], 2016 (DUAL 1 and 2 studies)	554; dcSSc 55.78%, mean age 50.4 years, mean disease duration 10.4 years	Macitentan: 3 mg *vs* 10 mg *vs* placebo for 16 weeks	Double-blind, placebo-controlled RCT	≥1 visible, active DU located at or distal to PIP that developed or worsened within 8 weeks and a history of additional DUs within 6–12 months of screening	Definition not stated but investigators provided with training to assess DUs prior to participating in study	Complete re-epithelialization of DU regardless of residual pain	CCB (42.06%) Pentoxifylline (16.97%) Antiplatelet agent (39.17%) Heparin (7.22%) ACEI (18.05%) ARB (9.38%) Glucocorticoids (53.79%) Immunosuppressants (53.07%)	Cumulative number of new DUs from baseline to week 16	No difference between groups—no reduction in the number of new DUs with treatment
Korn [19], 2004 (RAPIDS-1 study)	122; dcSSc 37.7%, mean age 51.8 years, mean disease duration 109.6 months	Bosentan: 62.5 mg BD for 4 weeks increased to 125 mg BD for 12 weeks	Prospective double-blind, placebo-controlled RCT	Documented DU within 12 months	Loss of surface epithelialization and not including fissures or cracks or areas of calcinotic extrusion, DU either at or distal to PIP	Complete healing: total re-epithelialization of all ulcers Partial healing: >50% reduction in surface area of DU	Oral vasodilating medication and oral medications for RP	Number of new DUs over 16 weeks	Reduced number of new ulcers with treatment 1.4/patient *vs* 2.7/patient (*P* < 0.01); no difference in healing rates
Matucci-Cerinic [18], 2011 (RAPIDS-2 study)	188; dcSSc 43.62%, mean age 49.5 years, mean disease duration 8.7 years	Bosentan: 62.5 mg BD for 4 weeks increased to 125 mg BD for remainder of study; total treatment duration 24 weeks + 8 week post-treatment follow-up period	Prospective double-blind, placebo-controlled RCT	At least 1 active DU (considered cardinal ulcer)	Painful area ≥2 mm in diameter with visible depth and loss of dermis, amenable to healing and in a location judged to be compatible with vascular lesion (volar surface of digit distal to PIP crease)	Complete epithelialization regardless of residual pain	CCB (52.66%) Anti-inflammatory or anti-rheumatic medication (36.70%) ACEI (15.96%) Peripheral vasodilators (11.70%) ARB (8.51%) Sildenafil (3.19%)	Mean number of new DUs and time to healing of cardinal ulcer	30% reduction of new DUs in treatment group (1.9 *vs* 2.7, *P* = 0.04) No difference in healing time
Prostacyclin analogues									
Seibold [36], 2017	148; dcSSc 35.14%, mean age 48.77 years, mean disease duration 10.5 years	Treprostinil (oral): 16 mg BD or maximally tolerated dose for 20 weeks	Double-blind, placebo-controlled RCT; randomization stratified by number of active DUs at baseline	At least one active DU at baseline	Area with visually discernible depth and a loss of continuity of epithelial coverage distal to PIP, volar to equator of finger and not localized to IP creases, triggered by trauma with no osteomyelitis or subtending calcinosis	Not stated	Background therapy for RP and DU permitted PDE5i (16.89%)	Change in net DU burden	Mean change in DU burden: treprostinil 0.43 *vs* placebo −0.10 (*P* = 0.20). No difference in percentage of patients who reached various threshold reductions of net DU burden. No effect on healing time or DU prevention
Wigley [31], 1994	126 (73 with digital lesions); mean age 48.9 years, mean disease duration: 8.65 years	Iloprost (i.v.): 5 consecutive days of 0.5–2 ng/kg/min for 6 h/day, 9 weeks follow-up	Double-blind, placebo-controlled RCT	SSc with RP	Digital lesions included ulcer, fissure or paronychia	Reduction in the number of baseline finger lesions by at least 50%	No other vasodilator therapy permitted	Total number of RP attacks, duration of RP attack and RP severity score	No significant difference between number of patients improved over 9 week period (*P* > 0.05)
Vayssairat [37], 1999	107; mean age 52 years, mean disease duration 5.9 years	Beraprost sodium (oral): 60 μg TDS for 6 months	Double-blind, placebo-controlled RCT; block randomization	History of DU within 3 years that healed at least 1 month before inclusion	Not stated	Not stated	Usual care with CCB, corticosteroids, D-penicillamine, colchicine continued	Percentage of patients with new DU	New DUs 48.1% (treatment) *vs* 58.8% (placebo) of patients (*P* = 0.325) Median time to DU: 160 days (treatment) *vs* 105 (placebo) (*P* = 0.233
Denton [38], 2017	74 (14.9% had DU at baseline); dcSSc 35.1%, mean age 52.6 years, mean disease duration 9.0 years	Selexipag: 1600 μg BD or maximally tolerated dose; 2–4 week single-blind placebo run-in, 8 week treatment, 30 day post-treatment follow-up	Double-blind, placebo-controlled RCT	≥7 RP attacks on ≥5 different days during baseline week and ≥80% eDiary compliance	Not stated	Not stated	CCB 52.7% Previous treatment for SSc and RP permitted, including ERAs No prostacyclin analogue permitted	Weekly average RP attacks Secondary endpoint: number of new DUs and number of baseline DUs completely healed at week 8	0.2 new DUs per patient in placebo group *vs* 0.4 in treatment group, all baseline DUs in treatment group healed, 5/8 baseline DUs in placebo group healed (not significant)
Kawald [39], 2008	50; dcSSc 30%, mean age 50.65 years, mean disease duration 4.55 years	Iloprost (i.v.): high (0.5–2.0 ng/kg/min) *vs* low (0.5 ng/kg/min) dose for 21 days once or twice per year	Randomised, open-label, placebo-controlled study	Severe RP or new active DU distal to MCP	Loss of both epidermis and dermis in the area of at least 2 mm diameter at distal phalanx	Not stated	CCB (52%) ACEI (20%) Pentoxifylline (30%)	Healing of DU	No significant difference between groups. High *vs* low dose: 63 *vs* 64 DU pre-treatment decreased to 15 *vs* 25 DU post-treatment
Wigley [29], 1992	35 (11 with DUs); dcSSc 37.14%, mean age 46.67 years	Iloprost (i.v.): 5 consecutive days, 0.5–2.0 ng/kg/min infusion for 6 h daily Average dose 1⋅32 ng/kg/min 10 weeks follow up	Double-blind, placebo-controlled RCT	≥8 symptomatic RP episodes per week without vasoactive treatment	Digital cutaneous lesions included ulcers, fissures and paronychia	Healing of all lesions observed at baseline	Corticosteroids, immunosuppressants, D-penicillamine, NSAIDs permitted	Daily RP diary—number, duration, and severity of attacks Effect on cutaneous lesions Haemodynamic effects	6 (86%) *vs* 0 (0%) patients had complete healing of digital lesions with treatment (*P* = 0.015) 4 (100%) *vs* 0 (0%) patients with DUs had complete healing of DUs with treatment (*P* = 0.029) 11 *vs* 29 new DUs in 10 weeks of follow-up in treatment *vs* placebo group
Antiplatelet agents									
Beckett [40], 1984	41; disease onset within 3 years	Dipyridamole/Aspirin: 75 mg/300 mg TDS Follow-up for up to 2 years	Double-blind, placebo controlled, RCT Randomization stratified according to age <30 or >30 years and organ involvement	Onset of SSc symptoms within 3 years	Finger ulcers	Not stated	Antihypertensive (9.75%) Corticosteroids (4.87%)	Improvement or worsening of general SSc	No benefit from treatment. No change in DU status over follow-up
Statins									
Abou-Raya [42], 2008	84; dcSSc 27.38%, mean age: 48.6 years, mean disease duration 6.7 years	Atorvastatin: 40 mg daily for 4 months	Double-blind, placebo-controlled study	RP and history of DUs within past 12 months despite vasodilator therapy	Loss of surface epithelium at or distal to PIP	Not stated	CCB (87%) ACEI (48%) ARB (21%) Aspirin (48%) Pentoxyphylline (44%) Corticosteroids (29%)	Vascular function and endothelial injury	−26% change in the number of DUs (*P* < 0.001) in favour of statin, mean number of DUs per patient 1.6 *vs* 2.5 (*P* = 0.003) in favour of statin
Immunosuppression and other therapies									
Au [45], 2010	158 (12 patients with DU); dcSSc 59.5%, mean age 48.5 years, mean disease duration 3.1 years	Cyclophosphamide (oral): 2 mg/kg as tolerated for 12 months	Double-blind, placebo-controlled RCT	Disease duration <7 years, ILD on HRCT	Digital dip ulcer distal to DIP joint	Not stated	Glucocorticoids (26.6%)	Number of patients with DU	No change in DU number over 52 weeks
Karalilova [44], 2021	66 (14 patients with DU); mean age 48.36 years, mean disease duration 34.62 years	Tofacitinib 5 mg BD *vs* methotrexate 10 mg/week; 52 weeks of follow-up	Pilot, randomized treatment study (single-blinded)	Definite SSc ≥24 weeks duration	Not stated	Not stated	Prednisolone (9.09%) Vasodilatory therapy not reported	Change in mRSS and safety data	Tofacitinib group: 75% reduction in total DU count Methotrexate group: 15% increase in total DU count
Nagaraja [51], 2019	17; dcSSc 47%, mean age 51 years, mean disease duration: 10.4 years	Riociguat: 2.5 mg TDS or maximally tolerated dose, for 16 weeks + 16 week open-label extension phase for participants with active DU or recurrence of DU at end of treatment	Double-blind, randomized, proof-of-concept trial, placebo controlled	At least 1 visible active ischaemic DU or painful indeterminate DU distal to PIP that developed or worsened within 8 weeks prior	Active DU: full-thickness skin lesion >3 mm in maximum diameter with loss of epithelization, epidermis and dermis Indeterminate DU: denudation could not be visualised and no other features of activity	Not stated	Vasodilators (12%) Corticosteroids (6%)	Change from baseline to week 16 net ulcer burden (total number of DUs at assessment) Designed to capture cumulative ulcer burden	No significant difference with riociguat treatment for DU

ACEI: angiotensin-converting enzyme inhibitor; ADP: adenosine diphosphate; ARB: angiotensin receptor antagonist; BD: twice daily; CREST: calcinosis, Raynaud’s phenomenon, oesophageal dysmotility, sclerodactyly, telangiectasia syndrome; HR: hazard ratio; IP: interphalangeal; mRSS: modified Rodnan Skin Score; NS: not significant; PAH: pulmonary arterial hypertension; PVR: peripheral vascular resistance; TDS: three times daily.

a Study included one patient with mixed connective tissue disease.

Only studies that recruited patients with definite SSc, generally according to either the 1980 ACR criteria [54] or 2013 ACR/EULAR classification criteria for SSc [55], were included in the review. The only study that evaluated the efficacy of tadalafil presented the results of one patient with MCTD along with those of the patients with SSc [9]. Given the significant phenotypic overlap of patients with MCTD and SSc and this was the only study available evaluating tadalafil, the results of this study were included.

Twenty-six (55%) studies specified a definition of DU. Of these studies, two early studies included skin fissures in the study definition [28, 29]. There was no consistent definition of DU applied across all studies, however, a DU was commonly considered to be a loss of surface epithelialization. The location of ulcers most commonly specified was a lesion at or distal to the proximal interphalangeal joint, most commonly on the volar aspect of the hand, and in more recent studies, specification that the lesion not be located over areas of calcification. The location of DUs and identification of areas of calcification were the only strategies applied in studies to attempt to identify ischaemic DU. The reliability of such methods was not tested in any included study. The location of DUs was not consistently reported across all studies. A healed DU was consistently reported to be present once complete re-epithelialization had occurred [10, 15–20, 30].

### CCBs

Two small studies (one RCT and one OBS) with a total of 13 patients suggested a reduction in the number of DUs with nifedipine [7, 8]. Neither study specified a definition of DU. Headache and dizziness, flushing and peripheral oedema were all reported as side effects of CCB therapy [7, 8].

### PDE5 inhibitor

Accelerated complete healing of active DUs with sildenafil was demonstrated in one small RCT at a moderate RoB, however, a definition of DU or ulcer healing was not stated along with the study results [11]. Two placebo-controlled RCTs with a clear definition of DU but at a moderate RoB [9, 10] demonstrated a reduction in the number of new DUs. Results of OBSs, all at a moderate RoB, indicated improved healing of DUs with sildenafil [12–14]. However, treatment efficacy across all uncontrolled OBSs was difficult to determine due to the high use of vasoactive concomitant medications such as CCBs, PDE5is and i.v. iloprost, which have either demonstrated or hypothesized efficacy in the treatment of DUs. Between 0 and 12% of patients ceased treatment with PDE5i (Table 3) [9–13]. The most common side effects of PDE5i were headache, flushing, pain, nasopharyngitis, palpitations, upper gastrointestinal tract symptoms and peripheral oedema [9–13, 15].

**Table 3.** Adverse events from systemic pharmacological treatment of SSc DUs^a^

Treatment	Headache/dizziness, %	Flushing, %	Pain, %	Nasopharyngitis, %	Paraesthesia, %	Palpitations, %	Upper GI symptoms^b^, %	Diarrhoea, %	Peripheral oedema, %	Anaemia, %	Elevated LFTs, %	Infection, %
CCB	11–50 [7, 8]	50 [7]	NR	NR	NR	NR	NR	NR	75 [7]	NR	NR	NR
PDE5i	1–38 [9–11, 13]	8–19 [9, 11, 12, 15]	13 [9]	29 [9]	NR	9–25 [12, 15]	9–13 [11, 12]	NR	9–19 [12, 15]	NR	NR	NR
ERA	2–20 [15, 16, 20, 23]	12–50 [15, 16]	6–7 [17, 19]	10–13 [20, 23]	30 [16]	NR	20 [16]	9–11 [17, 19, 20]	10–75 [15–18, 20, 26]	4–18 [15, 17, 20, 23, 24]	4–43 [15–21, 23, 24, 27]	NR
Prostacyclin analogues	12–100 [29, 31, 36–39]	11–50 [29, 31, 36, 38, 39]	11–23 [31, 36, 38]	NR	NR	NR	4–78 [29, 31, 36, 38, 39]	20–52 [31, 36, 38]	NR	NR	NR	NR
Antiplatelet agents	NR	NR	NR	NR	NR	NR	5 [40]	NR	NR	NR	NR	NR
N-acetylcysteine	2–23 [48, 49]	4–14 [48, 49]	14 [49]	NR	14 [49]	NR	NR	5 [49]	9 [49]	NR	NR	NR
Baricitinib	NR	NR	NR	NR	NR	NR	NR	NR	NR	NR	NR	10 (*n* = 1) [50]
Methotrexate	NR	NR	NR	NR	NR	NR	NR	NR	NR	NR	6.06 [44]	NR

a Data are presented for therapies where treatment-related adverse events were reported.

b Upper GI symptoms: nausea, vomiting and reflux.

GI: gastrointestinal; LFTs: liver function tests; NR: not reported.

### ERAs

Effective prevention of new DUs with bosentan, as compared with placebo, has been demonstrated in two RCTs, with careful attention paid to the clinical definition of DU in both studies [18, 19]. Of note, this finding was not replicated in two RCTs of macitentan compared with placebo, albeit in a milder DU population with a much lower new ulcer rate in the placebo patients than in the pivotal trials of bosentan [20]. Observational data of low to high RoB has supported this finding, with long-term bosentan treatment being associated with a reduction in the overall number of DUs, including in those patients with DUs despite treatment with i.v. iloprost [17, 21–24, 53]. OBSs have suggested improved healing of DUs with bosentan [16, 17, 21–25] and ambrisentan [26, 27], however, results of four RCTs with a moderate RoB showed that ERAs have no effect on healing rates of active DUs [18–20]. Observational data at a moderate RoB compared ERA therapy with PDE5i, showing no difference in time to healing with either treatment, but a reduced risk of development of new DUs with ERA as compared with PDE5i treatment [15]. Treatment discontinuation due to adverse events occurred in 4–20% of patients across both RCTs and OBSs of ERAs (Table 3) [9, 10, 12, 18, 20, 21, 51]. Headache, flushing, nausea, peripheral oedema and abnormal liver function tests (LFTs) with ERA therapy were the most commonly reported adverse events. All changes in LFTs were reversible with the withdrawal of therapy [15–21, 23, 24, 27].

### Prostacyclin analogues

No study has evaluated i.v. iloprost compared with placebo with DU healing as a primary endpoint. Two RCTs at a moderate RoB have evaluated i.v. iloprost for the management of RP and assessed DU healing as a secondary endpoint, with inconsistent application of definitions of DU and other finger lesions applied between studies. One study that included 11 patients at baseline with DUs showed improved DU healing in the treatment arm [29]. A follow-up study that included 73 participants with baseline digital cutaneous lesions (both DUs and skin fissures) failed to show a significant improvement in DU healing over 9 weeks [31]. Observational data, rated as having moderate to high RoB, suggests an improvement in DU healing following treatment with oral or i.v. prostacyclin analogues [28, 32–35]. However, no observational study included an appropriate control group, therefore true treatment efficacy is difficult to determine. No placebo-controlled RCT of oral prostacyclin analogues has shown treatment efficacy of this class of medication [36–38]. One small study, at high RoB, compared the direct costs of treating refractory DUs with i.v. prostaglandin analogues compared with botulinum toxin A injections [30]. The costs of the systemic i.v. therapy was significantly higher than those of local therapy with botulinum toxin A injections. The efficacy of both medications was not directly compared, so it is not possible to draw conclusions as to whether the clinical efficacy of either therapy is superior to further inform any cost-effectiveness analysis. Adverse effects of treatment were a near-universal finding in patients treated with prostacyclin analogues, however, they led to treatment cessation in a minority (3–24%) of patients [29, 32, 34, 37, 38]. A serious adverse event of ongoing clinical consequence was reported in one study—a central retinal vein thrombosis that was possibly attributed to iloprost therapy [34]. All other documented adverse events due to prostacyclin analogue therapy, such as such as headache, dizziness, flushing and gastrointestinal symptoms, were reversible upon cessation of the medication.

### Antiplatelet agents

No change in DU burden was observed in a placebo-controlled RCT of dipyramidole and aspirin [40], and one pilot observational study of clopidogrel suggested there may be an increased rate of endothelial dysfunction and DUs with clopidogrel use [41]. Upper gastrointestinal symptoms were reported in a single study as a result of treatment, otherwise antiplatelet agents were well tolerated [40].

### Statins

One RCT at a moderate RoB showed improved healing with the use of atorvastatin in addition to standard vasodilator therapy [42]. However, DUs were evaluated as a secondary endpoint in this study. No adverse events from statin use were reported in any study.

### Immunomodulatory and other agents

There has been no placebo-controlled RCTs of immunosuppressive therapies with DUs as a primary endpoint. The results of a small randomized study comparing the use of tofacitinib and low-dose oral methotrexate for treatment of SSc suggested tofacitinib was associated with a higher frequency of DU healing over 12 months [44]. A post hoc analysis of the Scleroderma Lung Study showed that cyclophosphamide as compared with placebo was not associated with a decrease in the number of DUs [45]. Observational data from four studies at a high RoB suggested improvement in DU healing with plasmapheresis [46, 47] and N-acetylcysteine infusions [48, 49]. One small study of riociguat, at a moderate RoB, applied a strict definition of DU but failed to show any treatment efficacy of riociguat and one-third of patients ceased treatment during the trial [51]. Adverse events were only variably reported in studies of immunomodulatory agents, however, infection was only rarely reported [50]. The use of N-acetylcysteine was associated with headache, flushing, pain, paraesthesia, diarrhoea and peripheral oedema [48, 49].

### Health-related quality of life (HRQoL) and patient-reported function outcomes

Twenty-two (47%) studies variably presented findings of the effects of DU treatment on patient HRQoL and function (Table 4). Ten studies reported changes in pain scores following successful DU treatment, with decreased pain scores recorded in 6 (60%) studies [12, 14, 27, 42]. Improved 36-item Short Form (SF-36) physical component scores were reported in one of the two studies that recorded SF-36 scores [9]. Positive effects on overall patient function with treatment of DUs was not commonly observed. Only 6 of 18 (33%) studies that reported global function demonstrated any improvement in global function with treatment of DUs. Despite effective prevention of new DUs, improved function was not observed in either of the bosentan RCTs [18, 19]. Half of the patients who achieved clinical control of active DUs in an observational study had a clinically significant improvement in function after 12 months of treatment with bosentan [21]. However, this finding was not observed in any randomized studies of bosentan [18, 19]. Three further studies showed a statistically significant improvement in HAQ Disability Index (HAQ-DI) scores (one each for PDE5i, ERA and atorvastatin) [9, 14, 42], but the change did not reach the threshold of minimal clinically important difference of the HAQ-DI [56, 57].

**Table 4.** Summary of reported HRQoL and functional outcomes

Therapy/study	HRQoL			Function	
	Pain	SF-36	Other	HAQ-DI	Other
PDE5i					
Hachulla [10]	No change	NR	NR	No change	No change in CHFS
Shenoy [9]	Improved	Improved	NR	Improved	NR
Brueckner [12]	Improved	NR	NR	No change	Improved VAS for daily activity
Della Rossa [14]	Improved	NR	NR	Improved	NR
ERA					
Khanna [20]	No change	NR	NR	No change	NR
Korn [19]	NR	NR	NR	No change^a^	NR
Matucci-Cerinic [18]	No change	NR	NR	No change	NR
Mouthon [21]	NR	No change	NR	Improved	CHFS improved
Kucuksahin [25]	NR	NR	NR	Improved	NR
de la Pena-Lefebvre [23]	NR	NR	NR	No change	Improved hand flexion
Kuhn [16]	NR	NR	NR	No change	NR
Parisi [27]	Improved	NR	NR	No change	NR
Prostacyclin analogues					
Seibold [36]	No change	NR	NR	No change	CHFS improved
Wigley [31]	NR	NR	NR	NR	No change MSHAQ
Vayssairat [37]	NR	NR	Improved overall well being	NR	NR
Bettoni [34]	NR	NR	NR	No change	NR
Shenavandeh [30]	Improved	NR	NR	NR	NR
Antiplatelet agents					
Ntelis [41]	NR	NR	NR	No change	NR
Atorvastatin					
Abou-Raya [42]	Improved	NR	NR	Improved	NR
Kuwana [43]	NR	NR	No change AIMS2	No change	NR
Riociguat					
Nagaraja [51]	NR	NR	No change PROMIS-29	No change	NR
Baricitinib					
Hou [50]	NR	NR	Improved overall disease severity	NR	Reduced DU impact

a Individual HAQ component scores and hand function improved in treatment group, no change in overall HAQ-DI scores.

AIMS2: Arthritis Impact Measurement Scales; CHFS: Cochin hand function score; MSHA: Modified Stanford Health Assessment Questionnaire; NR: not reported; SHAQ-DI: Scleroderma Health Assessment Questionnaire Disability Index; VAS: visual analogue scale.

## Discussion

A key finding of our SLR is that while treatment of active DUs was shown to be successful using iloprost, PDE5i and perhaps atorvastatin, the data are not very robust. There is stronger evidence for the impact of systemic pharmacological therapies for prevention compared with the treatment of active DUs. Data from both RCTs and OBSs support the use of ERAs, particularly bosentan, for the prevention of future DUs. There is an absence of robust evidence from large placebo-controlled RCTs to support the use of any systemic therapy for the treatment of active DUs. Small trials have shown the efficacy of i.v. iloprost, PDE5i and atorvastatin for the management of active DUs. However, the role of statins in the management of SSc DUs remains unresolved, as atorvastatin has not been evaluated in a study with DUs as a primary endpoint and previous data have shown no positive effect from atorvastatin on peripheral blood flow in patients with SSc [58]. Additional observational data suggest that CCBs, plasmapheresis, N-acetylcysteine and ketanserin may have a role in the management of active DUs. The strength of any recommendation to support the use of i.v. iloprost is limited by an absence of data from dedicated RCTs adequately powered to evaluate treatment efficacy for this therapy for DUs rather than RP. Much of the data used to justify various treatment strategies for SSc DUs are drawn from studies of RP that are underpowered to truly demonstrate treatment efficacy for DU outcomes. Although CCBs are generally recommended as a first-line agent in the management of digital vasculopathy in SSc [59], there is a paucity of evidence regarding the role CCBs in the prevention and/or treatment of DUs.

Future dedicated studies of management strategies for SSc DUs, with DU endpoints as their primary outcomes, are needed to develop a stronger evidence base to inform the management of active DUs. Newer agents are commonly ‘added on’ to an undefined standard of care. However, it is arguable there is no established standard of care for management of SSc DUs, nor is there evidence to support this add-on therapeutic strategy. A major challenge remains the interpretation of true treatment effect of any agent under investigation, given the confounding effects of background systemic and local therapies. The role of combination therapy or ‘step-up’ treatment, akin to the treat-to-target approach applied to many other inflammatory rheumatological conditions, is little studied. Currently there have been no head-to-head trials to demonstrate superior efficacy or the equivalence of one treatment compared with another. Future studies are also required to better understand the relationship between a reduction in burden of DUs and patient function and formal health economic analysis is required to establish the cost-effectiveness of various DU treatment strategies. Our results have shown that reduced DU burden, indicated by either improved healing or prevention of further DUs, is associated with an improvement in pain, but does not necessarily correlate with improved function.

To date, studies have not examined the impact on the non-pharmacological management of DUs, including lifestyle modifications such as smoking cessation. The contribution of smoking and macrovascular disease with DUs remains unresolved, with conflicting results from observational data [60–64]. The role of aggressive management of important comorbidities such as cardiovascular disease and diabetes mellitus as an adjunct to specific SSc DU therapies is undefined. Local pharmaceutical therapies in addition to local surgical procedures may also offer therapeutic benefit in the management of SSc DUs and is the topic of another SLR. A recent consensus statement from the Arthritis and Collaboration Hub Study Group highlighted both the importance to patients of understanding these management strategies and the absence of high-quality evidence to guide treatment decisions in the management of peripheral vascular manifestations of SSc [65].

The search methodology employed In this study was consciously inclusive to capture as many studies as possible evaluating systemic therapies for SSc DUs. However, only studies published in the English language were evaluated and therefore it is possible that pertinent studies published in other languages were excluded. The significant heterogeneity among study methodologies and outcome measures used limits the comparisons that can be drawn between multiple studies. There is yet to be a universally adopted definition of DU in clinical studies or a definition of what constitutes DU healing. It is critical that a specific and universal definition of ‘active’ DU is applied across studies and a careful distinction is made between ischaemic, traumatic and calcinotic ulcers. Vasoactive medications are unlikely to be effective in the healing of non-ischaemic ulcers. Therefore, if non-ischaemic ulcer types are not excluded from clinical trials, the lack of success of novel therapeutic strategies may be the result of study methodological limitations and inappropriate participant recruitment rather than true inefficacy of a novel treatment.

Furthermore, there remains a lack of consensus as to the optimal study duration and primary endpoints of DU RCTs required to establish treatment efficacy. Frequently, methods for randomization pertaining to DUs were unreported and it was unclear whether DU outcomes were analysed according to prespecified statistical analyses. Clinical experience indicates that healing of individual DUs can be slow and any study that aims to measure therapeutic efficacy over a period of weeks may be too brief a time period to demonstrate any treatment effect. Improvement in pain management and hand function remain important clinical outcomes for patients, and the absence of long-term observation following therapeutic intervention has resulted in a knowledge gap regarding the long-term symptomatic and functional benefits, or otherwise, of pharmacological intervention for SSc DUs. Arguably, an improved understanding of the natural history of DUs and, in particular, the expected time to healing of individual lesions may provide the rationale for a longer duration of future therapeutic studies. Longer treatment duration and longer trial observation periods may provide much needed data to guide treatment decisions for active DUs.

The lack of standardization regarding background therapies and inclusion of local therapies likely confounds the results of the included studies, making it challenging to draw robust conclusions about the efficacy of any therapy. The challenge in measuring the treatment success of peripheral vascular manifestations of SSc is well recognized [66] and there are historical and ongoing efforts to develop robust outcome measures for clinical trial design, including a clinical definition of DU and RCT endpoints [67]. There remains limited evidence to draw upon to support the development of new treatment recommendations of SSc DUs and our study has highlighted areas for future research (Table 5).

**Table 5.** Research agenda

Development of validated outcome measures for SSc DUs, including definition of DU and healing of DU
Evaluation of the role of CCB in the management of SSc DUs
Study of i.v. prostacyclin analogues in a primary DU study
Evaluation of the efficacy of management of comorbidities in the treatment of SSc DUs (e.g. diabetes mellitus, smoking)
Non-pharmacological interventions for management of SSc DUs
Role of combination therapy or ‘step-up’ therapy in the management of active DUs
Evaluation of the effects of DU therapies on HRQoL and function
Further evaluation of the role of statins in the management of active DUs and prevention of new DUs
Cost-effectiveness analysis of DU therapies
Evaluation of effectiveness of DU therapies in prevention of DU complications

In conclusion, there is evidence from RCTs to support the use of i.v. iloprost, PDE5i and atorvastatin in the management of active DUs and bosentan for the prevention of future DUs. The use of Janus kinase inhibitors to manage DUs requires further investigation. These results will be used to support the development of the WSF-endorsed recommendations for the management of SSc DUs. However, there are important methodological limitations to many previous studies of SSc DUs, including the need to clearly define the clinical presentation of DUs, the lack of validated endpoints and that conclusions regarding new therapeutics are frequently drawn from analysis of trials’ secondary endpoints. Our data have highlighted areas of significant need for future research to improve our management of this important disease manifestation.

## Supplementary Material

kead289_Supplementary_Data

## Data Availability

The study search protocol and data dictionary are available with publication. Individual study data are not available.
