# Supplementary material for: Systemic pharmacological treatment of digital ulcers in systemic sclerosis: a systematic literature review
Source: Rheumatology (Oxford). 2023 Jun 19;62(12):3785–800. doi: 10.1093/rheumatology/kead289 (PMC10691932; doi:10.1093/rheumatology/kead289)
Supplement: kead289_Supplementary_Data [file kead289_supplementary_data.docx]

**Online supplementary material - Systemic pharmacological treatment of digital ulcers in systemic sclerosis: a systematic literature review**

**Table of contents**

|  | **Page** |
| --- | --- |
| **Supplementary Data S1:** Research questions and PICO | 2 |
| **Supplementary Data S2:** Search strategy | 3 |
| **Supplementary Data S3:**  List of studies excluded at full-text screening stage with reasons | 13 |
| **Supplementary Data S4:** Data extraction template | 17 |
| **Supplementary Table S1:** Characteristics of included observational studies of systemic pharmacological treatment of digital ulcers in systemic sclerosis and corresponding references | 19 |
| **Supplementary Table S2:** Risk of bias assessment of all included studies | 28 |

**Supplementary Data S1: Research questions and PICO**

- What are the main hierarchical principles that should guide a rheumatologist in managing the DU?
- What is the optimal systemic (pharmacological) treatment approach for DU prevention and/or healing in SSc?
- What is the safety of systemic treatment for SSc-DU?
- What is the efficacy of systemic treatment for SSc-DU?
- What treatment regimens/doses are used for systemic treatment of SSc-DU?
- Is there international variation in systemic treatment for DU?
- What are the costs of DU in SSc?
- Are there cost-savings associated with systemic treatment for DU?

**P: problem/population**

Patients with systemic sclerosis SSc (‘scleroderma’) and digital ulcers (‘scleroderma’)

**I: Intervention**

Systemic (pharmacological) treatment – oral or intravenous or alternative

**C: Comparison**

Standard of care, placebo, no comparator

**O: Outcome**

- Efficacy (DU prevention, DU healing: overall number of DUs and/or number of new DUs, DU pain,

DU complications (infection, gangrene, need for analgesia, need for hospitalisation, amputation))

-Safety (Treatment-emergent adverse events)

**Supplementary Data S2: Search strategy**

**PubMed**

<http://www.ncbi.nlm.nih.gov/pubmed?otool=leiden>

(("Scleroderma, Systemic"[Mesh] OR "Systemic Scleroderma"[tw] OR "SSc"[tw] OR "systemic sclerosis"[tw] OR "Diffuse Scleroderma"[tw] OR "Limited Scleroderma"[tw] OR "Scleroderma"[tw] OR "scleroderma*"[tw] OR "CREST syndrome"[tw]) AND ("digital ulcers"[tw] OR "digital ulcer"[tw] OR "digital ulcer*"[tw] OR (("Fingers"[Mesh] OR "Fingers"[tw] OR "Finger"[tw] OR "thumb"[tw] OR "Toes"[mesh] OR "toes"[tw] OR "toe"[tw] OR "hallux"[tw] OR "digital"[tw] OR "digit"[tw] OR "digits"[tw]) AND ("Skin Ulcer"[Mesh] OR "Ulcer"[tw] OR "Ulcers"[tw] OR "ulcer*"[tw]))) AND ("Systemic treatment"[tw] OR "Systemic treat*"[tw] OR "pharmacological treatment"[tw] OR "pharmacological treat*"[tw] OR "Systemic therapy"[tw] OR "Systemic therap*"[tw] OR "pharmacological therapy"[tw] OR "pharmacological therapy*"[tw] OR "Drug Therapy"[Mesh] OR "drug therapy"[Subheading] OR "Drug Therapy"[tw] OR "Vasodilator Agents"[Mesh] OR "Vasodilator Agents"[Pharmacological Action] OR "Vasodilators"[tw] OR "Vasodilator"[tw] OR "Prostaglandins"[Mesh] OR "Prostaglandins"[tw] OR "Prostaglandin"[tw] OR "Prostanoids"[tw] OR "Prostanoid"[tw] OR "Iloprost"[mesh] OR "Iloprost"[tw] OR "Ciloprost"[tw] OR "Ventavis"[tw] OR "Epoprostenol"[mesh] OR "Epoprostanol"[tw] OR "Prostaglandin I2"[tw] OR "Prostacyclin"[tw] OR "Veletri"[tw] OR "Epoprostenol Sodium"[tw] OR "Flolan"[tw] OR "treprostinil"[Supplementary Concept] OR "Treprostinil"[tw] OR "Orenitram"[tw] OR "Remodulin"[tw] OR "Phosphodiesterase 5 Inhibitors"[Mesh] OR "Phosphodiesterase 5 Inhibitors"[Pharmacological Action] OR "Phosphodiesterase 5 Inhibitors"[tw] OR "Phosphodiesterase 5 Inhibitor"[tw] OR "Sildenafil Citrate"[Mesh] OR "Sildenafil"[tw] OR "Revatio"[tw] OR "Homosildenafil"[tw] OR "Hydroxyhomosildenafil"[tw] OR "Viagra"[tw] OR "Acetildenafil"[tw] OR "Desmethylsildenafil"[tw] OR "Tadalafil"[Mesh] OR "Tadalafil"[tw] OR "Cialis"[tw] OR "Endothelin Receptor Antagonists"[Mesh] OR "Endothelin Receptor Antagonists"[Pharmacological Action] OR "Endothelin Receptor Antagonists"[tw] OR "Endothelin Receptor Antagonist"[tw] OR "Bosentan"[Mesh] OR "Bosentan"[tw] OR "Tracleer"[tw] OR "Calcium Channel Blockers"[Mesh] OR "Calcium Channel Blockers"[Pharmacological Action] OR "Calcium Channel Blockers"[tw] OR "Calcium Channel Blocker"[tw] OR "Nifedipine"[Mesh] OR "Nifedipine"[tw] OR "Cordipin"[tw] OR "Cordipine"[tw] OR "Corinfar"[tw] OR "Korinfar"[tw] OR "Nifangin"[tw] OR "Procardia"[tw] OR "Vascard"[tw] OR "Adalat"[tw] OR "Fenigidin"[tw] OR "Amlodipine"[Mesh] OR "Amlodipine"[tw] OR "Amlodis"[tw] OR "Astudal"[tw] OR "Norvasc"[tw] OR "Istin"[tw] OR "Amlor"[tw] OR "Acetylcysteine"[Mesh] OR "Acetylcysteine"[tw] OR "N acetylcysteine"[tw] OR "N Acetyl L cysteine"[tw] OR "Mercapturic Acid"[tw] OR "Solmucol"[tw] OR "Genac"[tw] OR "Acemuc"[tw] OR "Acetabs"[tw] OR "NAC AL"[tw] OR "Acetylcystein"[tw] OR "Acetyst"[tw] OR "Airbron"[tw] OR "Alveolex"[tw] OR "Bromuc"[tw] OR "Azubronchin"[tw] OR "Bisolvon NAC"[tw] OR "Broncho Fips"[tw] OR "BronchoFips"[tw] OR "Broncholysin"[tw] OR "Broncoclar"[tw] OR "Codotussyl"[tw] OR "Cystamucil"[tw] OR "Dampo Mucopect"[tw] OR "Mucopect, Dampo"[tw] OR "durabronchal"[tw] OR "Larylin NAC"[tw] OR "Eurespiran"[tw] OR "Exomuc"[tw] OR "Fluimucil"[tw] OR "NAC Zambon"[tw] OR "Fabrol"[tw] OR "Fluprowit"[tw] OR "Optipect Hustengetränk"[tw] OR "Muco Sanigen"[tw] OR "Frekatuss"[tw] OR "Jenacystein"[tw] OR "Jenapharm"[tw] OR "Lantamed"[tw] OR "Lindocetyl"[tw] OR "M Pectil"[tw] OR "MPectil"[tw] OR "mentopin Acetylcystein"[tw] OR "Muciteran"[tw] OR "Mucomyst"[tw] OR "Acetylin"[tw] OR "Mucosil"[tw] OR "Mucosol"[tw] OR "Mucosolvin"[tw] OR "Siccoral"[tw] OR "Siran"[tw] OR "Ilube"[tw] OR "Hoestil"[tw] OR "acebraus"[tw] OR "Anti-platelet therapy"[tw] OR "Platelet Aggregation Inhibitors"[Mesh] OR "Platelet Aggregation Inhibitors"[Pharmacological Action] OR "Platelet Aggregation Inhibitors"[tw] OR "Platelet Aggregation Inhibitor"[tw] OR "Aspirin"[Mesh] OR "Aspirin"[tw] OR "Acetylsalicylic Acid"[tw] OR "Acylpyrin"[tw] OR "Aloxiprimum"[tw] OR "Colfarit"[tw] OR "Dispril"[tw] OR "Easprin"[tw] OR "Ecotrin"[tw] OR "Endosprin"[tw] OR "Magnecyl"[tw] OR "Micristin"[tw] OR "Polopirin"[tw] OR "Polopiryna"[tw] OR "Solprin"[tw] OR "Solupsan"[tw] OR "Zorprin"[tw] OR "Acetysal"[tw] OR "Dipyridamole"[Mesh] OR "Dipyridamole"[tw] OR "Cerebrovase"[tw] OR "Persantine"[tw] OR "Persantin"[tw] OR "Curantil"[tw] OR "Curantyl"[tw] OR "Kurantil"[tw] OR "Miosen"[tw] OR "Novo Dipiradol"[tw] OR "Antistenocardin"[tw] OR "Cléridium"[tw] OR "Angiotensin-Converting Enzyme Inhibitors"[Mesh] OR "Angiotensin-Converting Enzyme Inhibitors"[Pharmacological Action] OR "Angiotensin-Converting Enzyme Inhibitors"[tw] OR "Angiotensin-Converting Enzyme Inhibitor"[tw] OR "ACE Inhibitors"[tw] OR "ACE Inhibitor"[tw] OR "(S)-malic acid 1'-O-beta-gentiobioside"[tw] OR "2-(2-(5-bromoindan-1-yl)-3-mercaptopropionylamino)-3-(1H-pyrrolo(2,3-b)pyridin-3-yl)propionic acid"[tw] OR "7-((2-(acetylthio)-1-oxo-3-methylpropyl)amino)-1,2,3,4,6,7,8,12b-octahydro-6-oxopyrido(2,1-a)(2)benzazepin-4-carboxyacid"[tw] OR "alacepril"[tw] OR "benazepril"[tw] OR "benazeprilat"[tw] OR "Captopril"[tw] OR "captopril, hydrochlorothiazide drug combination"[tw] OR "ceronapril"[tw] OR "Cilazapril"[tw] OR "cilazaprilat"[tw] OR "delapril"[tw] OR "Enalapril"[tw] OR "Enalaprilat"[tw] OR "Fosinopril"[tw] OR "fosinoprilat"[tw] OR "gemopatrilat"[tw] OR "glyceraldehyde 3-phosphate dehydrogenase (304-313)"[tw] OR "imidapril"[tw] OR "imidaprilat"[tw] OR "IRW peptide"[tw] OR "L-proline, N2-((1S)-1-carboxy-3-phenylpropyl)-N6-((4-hydroxyphenyl)iminomethyl)-L-lysyl-"[tw] OR "libenzapril"[tw] OR "Lisinopril"[tw] OR "LVV-hemorphin 6"[tw] OR "MDL 100240"[tw] OR "moexipril"[tw] OR "N-((1-((2-(acetylthio)-3-methyl-1-oxobutyl)amino)-1-cyclopentyl)carbonyl)-O-methyl-L-tyrosine ethyl ester"[tw] OR "N-(1-carboxy-3-phenylpropyl)-alanylalanine"[tw] OR "N-(2-(mercaptomethyl)-3-methylbutanoyl)-4-(1H-pyrazol-1-yl)phenylalanine"[tw] OR "omapatrilat"[tw] OR "Perindopril"[tw] OR "perindoprilat"[tw] OR "Quinapril"[tw] OR "quinaprilat"[tw] OR "Ramipril"[tw] OR "ramiprilat"[tw] OR "rentiapril"[tw] OR "sampatrilat"[tw] OR "spirapril"[tw] OR "temocapril hydrochloride"[tw] OR "Teprotide"[tw] OR "trandolapril"[tw] OR "valyl-prolyl-proline"[tw] OR "zofenopril"[tw] OR "Angiotensin Receptor Antagonists"[Mesh] OR "Angiotensin Receptor Antagonists"[Pharmacological Action] OR "Angiotensin Receptor Antagonists"[tw] OR "Angiotensin Receptor Antagonist"[tw] OR "Angiotensin Receptor Blockers"[tw] OR "Angiotensin Receptor Blocker"[tw] OR "1-Sarcosine-8-Isoleucine Angiotensin II"[tw] OR "2-(butyryl-(2'-(4,5-dimethylisoxazol-3-ylsulfamoyl)biphenyl-4-ylmethyl)amino)-N-isopropyl-3-methylbutyramide"[tw] OR "2-(butyryl-(2'-(4-chloro-5-methylisoxazol-3-ylsulfamoyl)biphenyl-4-ylmethyl)amino)-N-isopropyl-3-methylbutyramide"[tw] OR "2-(butyryl-(2'-(4-fluoro-5-methylisoxazol-3-ylsulfamoyl)biphenyl-4-ylmethyl)amino)-N-isopropyl-3-methylbutyramide"[tw] OR "2-butyl-3-((2'-(1H-tetrazol-5-yl)(1,1'-biphenyl)-4-yl)methyl)-2,3-diazaspiro(4.4)nonane-1,4-dione"[tw] OR "2-methylsulfanyl-3-(2'-(2H-tetrazol-5-yl)biphenyl-4-ylmethyl)-3H-quinazolin-4-one"[tw] OR "3-((2'-(benzoylaminosulfonyl)(1,1'-biphenyl)-4-yl)methyl)-2-butyl-2,3-diazaspiro(4.4)nonane-1,4-dione"[tw] OR "3-((2'-carboxybiphenyl-4-yl)methyl)-2-cyclopropyl-7-methyl-3H-imidazo(4,5-b)pyridine"[tw] OR "4'-((2-butyl-4-oxo-1,3-diazaspiro(4.4)non-1-en-3-yl)methyl)-N-(4,5-dimethyl-3-isoxazolyl)-2'-(ethoxymethyl)(1,1'-biphenyl)-2-sulfonamide"[tw] OR "57G709"[tw] OR "606A compound"[tw] OR "A 81988"[tw] OR "Abbott 81282"[tw] OR "Amlodipine Besylate, Olmesartan Medoxomil Drug Combination"[tw] OR "azilsartan medoxomil"[tw] OR "BMS 183920"[tw] OR "candesartan"[tw] OR "candesartan cilexetil"[tw] OR "dimethyl (1-methyl-1,3-benzimidazol-5-yl)aminomethylenepropanedioate"[tw] OR "enoltasosartan"[tw] OR "eprosartan"[tw] OR "GR 117289"[tw] OR "HN 65021"[tw] OR "Irbesartan"[tw] OR "KD3 671"[tw] OR "KR 31080"[tw] OR "KRH 594"[tw] OR "L 158809"[tw] OR "L 159913"[tw] OR "L 163017"[tw] OR "L 163082"[tw] OR "L 163958"[tw] OR "L 164282"[tw] OR "Losartan"[tw] OR "LR B-081"[tw] OR "N-(2-(6-((2-ethyl-5,7-dimethyl-3H-imidazo(4,5-b)pyridin-3-yl)methyl)quinolin-2-yl))trifluoromethanesulfonamide"[tw] OR "olmesartan"[tw] OR "Olmesartan Medoxomil"[tw] OR "PD 123319"[tw] OR "sacubitril-valsartan"[tw] OR "saprisartan potassium"[tw] OR "Saralasin"[tw] OR "SL 910102-90 DL"[tw] OR "tasosartan"[tw] OR "Telmisartan"[tw] OR "telmisartan amlodipine combination"[tw] OR "telmisartan, hydrochlorothiazide drug combination"[tw] OR "TH 142177"[tw] OR "UR 7247"[tw] OR "UR 7280"[tw] OR "Valsartan"[tw] OR "XR 510"[tw] OR "YM358"[tw] OR "ZD 7155"[tw] OR "Nitrates"[Mesh] OR "Nitrates"[tw] OR "Nitrate"[tw] OR "Tetranitrate"[tw] OR "Nicorandil"[tw] OR "Serotonin Uptake Inhibitors"[Mesh] OR "Serotonin Uptake Inhibitors"[Pharmacological Action] OR "Serotonin Uptake Inhibitors"[tw] OR "Serotonin Uptake Inhibitor"[tw] OR "Serotonin re uptake Inhibitors"[tw] OR "Serotonin re uptake Inhibitor"[tw] OR "Serotonin reuptake Inhibitors"[tw] OR "Serotonin reuptake Inhibitor"[tw] OR "Selective serotonin reuptake inhibitors"[tw] OR "Selective serotonin reuptake inhibitor"[tw] OR "Selective serotonin re uptake inhibitors"[tw] OR "Selective serotonin re uptake inhibitor"[tw] OR "SSRI"[tw] OR "SSRIs"[tw]))

**MEDLINE via OVID**

<http://gateway.ovid.com/ovidweb.cgi?T=JS&MODE=ovid&NEWS=n&PAGE=main&D=medall>

((exp "Scleroderma, Systemic"/ OR "Systemic Scleroderma".mp OR "SSc".mp OR "systemic sclerosis".mp OR "Diffuse Scleroderma".mp OR "Limited Scleroderma".mp OR "Scleroderma".mp OR "scleroderma*".mp OR "CREST syndrome".mp) AND ("digital ulcers".mp OR "digital ulcer".mp OR "digital ulcer*".mp OR ((exp "Fingers"/ OR "Fingers".mp OR "Finger".mp OR "thumb".mp OR "Toes"/ OR "toes".mp OR "toe".mp OR "hallux".mp OR "digital".mp OR "digit".mp OR "digits".mp) AND (exp "Skin Ulcer"/ OR "Ulcer".mp OR "Ulcers".mp OR "ulcer*".mp))) AND ("Systemic treatment".mp OR "Systemic treat*".mp OR "pharmacological treatment".mp OR "pharmacological treat*".mp OR "Systemic therapy".mp OR "Systemic therap*".mp OR "pharmacological therapy".mp OR "pharmacological therapy*".mp OR exp "Drug Therapy"/ OR "dt".fs OR "Drug Therapy".mp OR exp "Vasodilator Agents"/ OR "Vasodilators".mp OR "Vasodilator".mp OR exp "Prostaglandins"/ OR "Prostaglandins".mp OR "Prostaglandin".mp OR "Prostanoids".mp OR "Prostanoid".mp OR exp "Iloprost"/ OR "Iloprost".mp OR "Ciloprost".mp OR "Ventavis".mp OR exp "Epoprostenol"/ OR "Epoprostanol".mp OR "Prostaglandin I2".mp OR "Prostacyclin".mp OR "Veletri".mp OR "Epoprostenol Sodium".mp OR "Flolan".mp OR "treprostinil"/ OR "Treprostinil".mp OR "Orenitram".mp OR "Remodulin".mp OR exp "Phosphodiesterase 5 Inhibitors"/ OR "Phosphodiesterase 5 Inhibitors".mp OR "Phosphodiesterase 5 Inhibitor".mp OR exp "Sildenafil Citrate"/ OR "Sildenafil".mp OR "Revatio".mp OR "Homosildenafil".mp OR "Hydroxyhomosildenafil".mp OR "Viagra".mp OR "Acetildenafil".mp OR "Desmethylsildenafil".mp OR exp "Tadalafil"/ OR "Tadalafil".mp OR "Cialis".mp OR exp "Endothelin Receptor Antagonists"/ OR "Endothelin Receptor Antagonists".mp OR "Endothelin Receptor Antagonist".mp OR exp "Bosentan"/ OR "Bosentan".mp OR "Tracleer".mp OR exp "Calcium Channel Blockers"/ OR "Calcium Channel Blockers".mp OR "Calcium Channel Blocker".mp OR exp "Nifedipine"/ OR "Nifedipine".mp OR "Cordipin".mp OR "Cordipine".mp OR "Corinfar".mp OR "Korinfar".mp OR "Nifangin".mp OR "Procardia".mp OR "Vascard".mp OR "Adalat".mp OR "Fenigidin".mp OR exp "Amlodipine"/ OR "Amlodipine".mp OR "Amlodis".mp OR "Astudal".mp OR "Norvasc".mp OR "Istin".mp OR "Amlor".mp OR exp "Acetylcysteine"/ OR "Acetylcysteine".mp OR "N acetylcysteine".mp OR "N Acetyl L cysteine".mp OR "Mercapturic Acid".mp OR "Solmucol".mp OR "Genac".mp OR "Acemuc".mp OR "Acetabs".mp OR "NAC AL".mp OR "Acetylcystein".mp OR "Acetyst".mp OR "Airbron".mp OR "Alveolex".mp OR "Bromuc".mp OR "Azubronchin".mp OR "Bisolvon NAC".mp OR "Broncho Fips".mp OR "BronchoFips".mp OR "Broncholysin".mp OR "Broncoclar".mp OR "Codotussyl".mp OR "Cystamucil".mp OR "Dampo Mucopect".mp OR "Mucopect, Dampo".mp OR "durabronchal".mp OR "Larylin NAC".mp OR "Eurespiran".mp OR "Exomuc".mp OR "Fluimucil".mp OR "NAC Zambon".mp OR "Fabrol".mp OR "Fluprowit".mp OR "Optipect Hustengetränk".mp OR "Muco Sanigen".mp OR "Frekatuss".mp OR "Jenacystein".mp OR "Jenapharm".mp OR "Lantamed".mp OR "Lindocetyl".mp OR "M Pectil".mp OR "MPectil".mp OR "mentopin Acetylcystein".mp OR "Muciteran".mp OR "Mucomyst".mp OR "Acetylin".mp OR "Mucosil".mp OR "Mucosol".mp OR "Mucosolvin".mp OR "Siccoral".mp OR "Siran".mp OR "Ilube".mp OR "Hoestil".mp OR "acebraus".mp OR "Anti-platelet therapy".mp OR exp "Platelet Aggregation Inhibitors"/ OR "Platelet Aggregation Inhibitors".mp OR "Platelet Aggregation Inhibitor".mp OR "Aspirin"/ OR "Aspirin".mp OR "Acetylsalicylic Acid".mp OR "Acylpyrin".mp OR "Aloxiprimum".mp OR "Colfarit".mp OR "Dispril".mp OR "Easprin".mp OR "Ecotrin".mp OR "Endosprin".mp OR "Magnecyl".mp OR "Micristin".mp OR "Polopirin".mp OR "Polopiryna".mp OR "Solprin".mp OR "Solupsan".mp OR "Zorprin".mp OR "Acetysal".mp OR exp "Dipyridamole"/ OR "Dipyridamole".mp OR "Cerebrovase".mp OR "Persantine".mp OR "Persantin".mp OR "Curantil".mp OR "Curantyl".mp OR "Kurantil".mp OR "Miosen".mp OR "Novo Dipiradol".mp OR "Antistenocardin".mp OR "Cléridium".mp OR exp "Angiotensin-Converting Enzyme Inhibitors"/ OR "Angiotensin-Converting Enzyme Inhibitors".mp OR "Angiotensin-Converting Enzyme Inhibitor".mp OR "ACE Inhibitors".mp OR "ACE Inhibitor".mp OR "(S)-malic acid 1'-O-beta-gentiobioside".mp OR "2-(2-(5-bromoindan-1-yl)-3-mercaptopropionylamino)-3-(1H-pyrrolo(2,3-b)pyridin-3-yl)propionic acid".mp OR "7-((2-(acetylthio)-1-oxo-3-methylpropyl)amino)-1,2,3,4,6,7,8,12b-octahydro-6-oxopyrido(2,1-a)(2)benzazepin-4-carboxyacid".mp OR "alacepril".mp OR "benazepril".mp OR "benazeprilat".mp OR "Captopril".mp OR "captopril, hydrochlorothiazide drug combination".mp OR "ceronapril".mp OR "Cilazapril".mp OR "cilazaprilat".mp OR "delapril".mp OR "Enalapril".mp OR "Enalaprilat".mp OR "Fosinopril".mp OR "fosinoprilat".mp OR "gemopatrilat".mp OR "glyceraldehyde 3-phosphate dehydrogenase (304-313)".mp OR "imidapril".mp OR "imidaprilat".mp OR "IRW peptide".mp OR "L-proline, N2-((1S)-1-carboxy-3-phenylpropyl)-N6-((4-hydroxyphenyl)iminomethyl)-L-lysyl-".mp OR "libenzapril".mp OR "Lisinopril".mp OR "LVV-hemorphin 6".mp OR "MDL 100240".mp OR "moexipril".mp OR "N-((1-((2-(acetylthio)-3-methyl-1-oxobutyl)amino)-1-cyclopentyl)carbonyl)-O-methyl-L-tyrosine ethyl ester".mp OR "N-(1-carboxy-3-phenylpropyl)-alanylalanine".mp OR "N-(2-(mercaptomethyl)-3-methylbutanoyl)-4-(1H-pyrazol-1-yl)phenylalanine".mp OR "omapatrilat".mp OR "Perindopril".mp OR "perindoprilat".mp OR "Quinapril".mp OR "quinaprilat".mp OR "Ramipril".mp OR "ramiprilat".mp OR "rentiapril".mp OR "sampatrilat".mp OR "spirapril".mp OR "temocapril hydrochloride".mp OR "Teprotide".mp OR "trandolapril".mp OR "valyl-prolyl-proline".mp OR "zofenopril".mp OR exp "Angiotensin Receptor Antagonists"/ OR "Angiotensin Receptor Antagonists".mp OR "Angiotensin Receptor Antagonist".mp OR "Angiotensin Receptor Blockers".mp OR "Angiotensin Receptor Blocker".mp OR "1-Sarcosine-8-Isoleucine Angiotensin II".mp OR "2-(butyryl-(2'-(4,5-dimethylisoxazol-3-ylsulfamoyl)biphenyl-4-ylmethyl)amino)-N-isopropyl-3-methylbutyramide".mp OR "2-(butyryl-(2'-(4-chloro-5-methylisoxazol-3-ylsulfamoyl)biphenyl-4-ylmethyl)amino)-N-isopropyl-3-methylbutyramide".mp OR "2-(butyryl-(2'-(4-fluoro-5-methylisoxazol-3-ylsulfamoyl)biphenyl-4-ylmethyl)amino)-N-isopropyl-3-methylbutyramide".mp OR "2-butyl-3-((2'-(1H-tetrazol-5-yl)(1,1'-biphenyl)-4-yl)methyl)-2,3-diazaspiro(4.4)nonane-1,4-dione".mp OR "2-methylsulfanyl-3-(2'-(2H-tetrazol-5-yl)biphenyl-4-ylmethyl)-3H-quinazolin-4-one".mp OR "3-((2'-(benzoylaminosulfonyl)(1,1'-biphenyl)-4-yl)methyl)-2-butyl-2,3-diazaspiro(4.4)nonane-1,4-dione".mp OR "3-((2'-carboxybiphenyl-4-yl)methyl)-2-cyclopropyl-7-methyl-3H-imidazo(4,5-b)pyridine".mp OR "4'-((2-butyl-4-oxo-1,3-diazaspiro(4.4)non-1-en-3-yl)methyl)-N-(4,5-dimethyl-3-isoxazolyl)-2'-(ethoxymethyl)(1,1'-biphenyl)-2-sulfonamide".mp OR "57G709".mp OR "606A compound".mp OR "A 81988".mp OR "Abbott 81282".mp OR "Amlodipine Besylate, Olmesartan Medoxomil Drug Combination".mp OR "azilsartan medoxomil".mp OR "BMS 183920".mp OR "candesartan".mp OR "candesartan cilexetil".mp OR "dimethyl (1-methyl-1,3-benzimidazol-5-yl)aminomethylenepropanedioate".mp OR "enoltasosartan".mp OR "eprosartan".mp OR "GR 117289".mp OR "HN 65021".mp OR "Irbesartan".mp OR "KD3 671".mp OR "KR 31080".mp OR "KRH 594".mp OR "L 158809".mp OR "L 159913".mp OR "L 163017".mp OR "L 163082".mp OR "L 163958".mp OR "L 164282".mp OR "Losartan".mp OR "LR B-081".mp OR "N-(2-(6-((2-ethyl-5,7-dimethyl-3H-imidazo(4,5-b)pyridin-3-yl)methyl)quinolin-2-yl))trifluoromethanesulfonamide".mp OR "olmesartan".mp OR "Olmesartan Medoxomil".mp OR "PD 123319".mp OR "sacubitril-valsartan".mp OR "saprisartan potassium".mp OR "Saralasin".mp OR "SL 910102-90 DL".mp OR "tasosartan".mp OR "Telmisartan".mp OR "telmisartan amlodipine combination".mp OR "telmisartan, hydrochlorothiazide drug combination".mp OR "TH 142177".mp OR "UR 7247".mp OR "UR 7280".mp OR "Valsartan".mp OR "XR 510".mp OR "YM358".mp OR "ZD 7155".mp OR exp "Nitrates"/ OR "Nitrates".mp OR "Nitrate".mp OR "Tetranitrate".mp OR "Nicorandil".mp OR exp "Serotonin Uptake Inhibitors"/ OR "Serotonin Uptake Inhibitors".mp OR "Serotonin Uptake Inhibitor".mp OR "Serotonin re uptake Inhibitors".mp OR "Serotonin re uptake Inhibitor".mp OR "Serotonin reuptake Inhibitors".mp OR "Serotonin reuptake Inhibitor".mp OR "Selective serotonin reuptake inhibitors".mp OR "Selective serotonin reuptake inhibitor".mp OR "Selective serotonin re uptake inhibitors".mp OR "Selective serotonin re uptake inhibitor".mp OR "SSRI".mp OR "SSRIs".mp))

**Embase**

<http://ovidsp.ovid.com/ovidweb.cgi?T=JS&PAGE=main&MODE=ovid&D=oemezd>

((exp "Scleroderma, Systemic"/ OR exp "systemic sclerosis"/ OR "Systemic Scleroderma".mp OR "SSc".mp OR "systemic sclerosis".mp OR "Diffuse Scleroderma".mp OR "Limited Scleroderma".mp OR "Scleroderma".mp OR "scleroderma*".mp OR "CREST syndrome".mp) AND ("finger ulcer"/ OR "digital ulcers".mp OR "digital ulcer".mp OR "digital ulcer*".mp OR ((exp "Fingers"/ OR exp "Finger"/ OR "Fingers".mp OR "Finger".mp OR "thumb".mp OR exp "Toes"/ OR exp "Toe"/ OR "toes".mp OR "toe".mp OR "hallux".mp OR "digital".mp OR "digit".mp OR "digits".mp) AND (exp "Skin Ulcer"/ OR "Ulcer".mp OR "Ulcers".mp OR "ulcer*".mp))) AND (exp "Systemic therapy"/ OR "Systemic treatment".mp OR "Systemic treat*".mp OR "pharmacological treatment".mp OR "pharmacological treat*".mp OR "Systemic therapy".mp OR "Systemic therap*".mp OR "pharmacological therapy".mp OR "pharmacological therapy*".mp OR exp "Drug Therapy"/ OR "dt".fs OR "Drug Therapy".mp OR exp "Vasodilator Agents"/ OR exp "Vasodilator Agent"/ OR "Vasodilators".mp OR "Vasodilator".mp OR exp "Prostaglandin"/ OR exp "Prostaglandins"/ OR "Prostaglandins".mp OR "Prostaglandin".mp OR "Prostanoids".mp OR "Prostanoid".mp OR exp "Iloprost"/ OR "Iloprost".mp OR "Ciloprost".mp OR "Ventavis".mp OR exp "Epoprostenol"/ OR "Epoprostanol".mp OR "Prostaglandin I2".mp OR "Prostacyclin".mp OR "Veletri".mp OR "Epoprostenol Sodium".mp OR "Flolan".mp OR "treprostinil"/ OR "Treprostinil".mp OR "Orenitram".mp OR "Remodulin".mp OR exp "Phosphodiesterase V Inhibitor"/ OR exp "Phosphodiesterase 5 Inhibitors"/ OR "Phosphodiesterase 5 Inhibitors".mp OR "Phosphodiesterase 5 Inhibitor".mp OR exp "Sildenafil Citrate"/ OR "Sildenafil".mp OR "Revatio".mp OR "Homosildenafil".mp OR "Hydroxyhomosildenafil".mp OR "Viagra".mp OR "Acetildenafil".mp OR "Desmethylsildenafil".mp OR exp "Tadalafil"/ OR "Tadalafil".mp OR "Cialis".mp OR exp "Endothelin Receptor Antagonist"/ OR exp "Endothelin Receptor Antagonists"/ OR "Endothelin Receptor Antagonists".mp OR "Endothelin Receptor Antagonist".mp OR exp "Bosentan"/ OR "Bosentan".mp OR "Tracleer".mp OR exp "calcium channel blocking agent"/ OR exp "Calcium Channel Blockers"/ OR "Calcium Channel Blockers".mp OR "Calcium Channel Blocker".mp OR exp "Nifedipine"/ OR "Nifedipine".mp OR "Cordipin".mp OR "Cordipine".mp OR "Corinfar".mp OR "Korinfar".mp OR "Nifangin".mp OR "Procardia".mp OR "Vascard".mp OR "Adalat".mp OR "Fenigidin".mp OR exp "Amlodipine"/ OR "Amlodipine".mp OR "Amlodis".mp OR "Astudal".mp OR "Norvasc".mp OR "Istin".mp OR "Amlor".mp OR exp "Acetylcysteine"/ OR "Acetylcysteine".mp OR "N acetylcysteine".mp OR "N Acetyl L cysteine".mp OR "Mercapturic Acid".mp OR "Solmucol".mp OR "Genac".mp OR "Acemuc".mp OR "Acetabs".mp OR "NAC AL".mp OR "Acetylcystein".mp OR "Acetyst".mp OR "Airbron".mp OR "Alveolex".mp OR "Bromuc".mp OR "Azubronchin".mp OR "Bisolvon NAC".mp OR "Broncho Fips".mp OR "BronchoFips".mp OR "Broncholysin".mp OR "Broncoclar".mp OR "Codotussyl".mp OR "Cystamucil".mp OR "Dampo Mucopect".mp OR "Mucopect, Dampo".mp OR "durabronchal".mp OR "Larylin NAC".mp OR "Eurespiran".mp OR "Exomuc".mp OR "Fluimucil".mp OR "NAC Zambon".mp OR "Fabrol".mp OR "Fluprowit".mp OR "Optipect Hustengetränk".mp OR "Muco Sanigen".mp OR "Frekatuss".mp OR "Jenacystein".mp OR "Jenapharm".mp OR "Lantamed".mp OR "Lindocetyl".mp OR "M Pectil".mp OR "MPectil".mp OR "mentopin Acetylcystein".mp OR "Muciteran".mp OR "Mucomyst".mp OR "Acetylin".mp OR "Mucosil".mp OR "Mucosol".mp OR "Mucosolvin".mp OR "Siccoral".mp OR "Siran".mp OR "Ilube".mp OR "Hoestil".mp OR "acebraus".mp OR "Anti-platelet therapy".mp OR exp "antithrombocytic agent"/ OR exp "Platelet Aggregation Inhibitors"/ OR "Platelet Aggregation Inhibitors".mp OR "Platelet Aggregation Inhibitor".mp OR "Aspirin"/ OR "Aspirin".mp OR "Acetylsalicylic Acid".mp OR "Acylpyrin".mp OR "Aloxiprimum".mp OR "Colfarit".mp OR "Dispril".mp OR "Easprin".mp OR "Ecotrin".mp OR "Endosprin".mp OR "Magnecyl".mp OR "Micristin".mp OR "Polopirin".mp OR "Polopiryna".mp OR "Solprin".mp OR "Solupsan".mp OR "Zorprin".mp OR "Acetysal".mp OR exp "Dipyridamole"/ OR "Dipyridamole".mp OR "Cerebrovase".mp OR "Persantine".mp OR "Persantin".mp OR "Curantil".mp OR "Curantyl".mp OR "Kurantil".mp OR "Miosen".mp OR "Novo Dipiradol".mp OR "Antistenocardin".mp OR "Cléridium".mp OR exp "dipeptidyl carboxypeptidase inhibitor"/ OR exp "Angiotensin-Converting Enzyme Inhibitors"/ OR "Angiotensin-Converting Enzyme Inhibitors".mp OR "Angiotensin-Converting Enzyme Inhibitor".mp OR "ACE Inhibitors".mp OR "ACE Inhibitor".mp OR "(S)-malic acid 1'-O-beta-gentiobioside".mp OR "2-(2-(5-bromoindan-1-yl)-3-mercaptopropionylamino)-3-(1H-pyrrolo(2,3-b)pyridin-3-yl)propionic acid".mp OR "7-((2-(acetylthio)-1-oxo-3-methylpropyl)amino)-1,2,3,4,6,7,8,12b-octahydro-6-oxopyrido(2,1-a)(2)benzazepin-4-carboxyacid".mp OR "alacepril".mp OR "benazepril".mp OR "benazeprilat".mp OR "Captopril".mp OR "captopril, hydrochlorothiazide drug combination".mp OR "ceronapril".mp OR "Cilazapril".mp OR "cilazaprilat".mp OR "delapril".mp OR "Enalapril".mp OR "Enalaprilat".mp OR "Fosinopril".mp OR "fosinoprilat".mp OR "gemopatrilat".mp OR "glyceraldehyde 3-phosphate dehydrogenase (304-313)".mp OR "imidapril".mp OR "imidaprilat".mp OR "IRW peptide".mp OR "L-proline, N2-((1S)-1-carboxy-3-phenylpropyl)-N6-((4-hydroxyphenyl)iminomethyl)-L-lysyl-".mp OR "libenzapril".mp OR "Lisinopril".mp OR "LVV-hemorphin 6".mp OR "MDL 100240".mp OR "moexipril".mp OR "N-((1-((2-(acetylthio)-3-methyl-1-oxobutyl)amino)-1-cyclopentyl)carbonyl)-O-methyl-L-tyrosine ethyl ester".mp OR "N-(1-carboxy-3-phenylpropyl)-alanylalanine".mp OR "N-(2-(mercaptomethyl)-3-methylbutanoyl)-4-(1H-pyrazol-1-yl)phenylalanine".mp OR "omapatrilat".mp OR "Perindopril".mp OR "perindoprilat".mp OR "Quinapril".mp OR "quinaprilat".mp OR "Ramipril".mp OR "ramiprilat".mp OR "rentiapril".mp OR "sampatrilat".mp OR "spirapril".mp OR "temocapril hydrochloride".mp OR "Teprotide".mp OR "trandolapril".mp OR "valyl-prolyl-proline".mp OR "zofenopril".mp OR exp "angiotensin receptor antagonist"/ OR exp "Angiotensin Receptor Antagonists"/ OR "Angiotensin Receptor Antagonists".mp OR "Angiotensin Receptor Antagonist".mp OR "Angiotensin Receptor Blockers".mp OR "Angiotensin Receptor Blocker".mp OR "1-Sarcosine-8-Isoleucine Angiotensin II".mp OR "2-(butyryl-(2'-(4,5-dimethylisoxazol-3-ylsulfamoyl)biphenyl-4-ylmethyl)amino)-N-isopropyl-3-methylbutyramide".mp OR "2-(butyryl-(2'-(4-chloro-5-methylisoxazol-3-ylsulfamoyl)biphenyl-4-ylmethyl)amino)-N-isopropyl-3-methylbutyramide".mp OR "2-(butyryl-(2'-(4-fluoro-5-methylisoxazol-3-ylsulfamoyl)biphenyl-4-ylmethyl)amino)-N-isopropyl-3-methylbutyramide".mp OR "2-butyl-3-((2'-(1H-tetrazol-5-yl)(1,1'-biphenyl)-4-yl)methyl)-2,3-diazaspiro(4.4)nonane-1,4-dione".mp OR "2-methylsulfanyl-3-(2'-(2H-tetrazol-5-yl)biphenyl-4-ylmethyl)-3H-quinazolin-4-one".mp OR "3-((2'-(benzoylaminosulfonyl)(1,1'-biphenyl)-4-yl)methyl)-2-butyl-2,3-diazaspiro(4.4)nonane-1,4-dione".mp OR "3-((2'-carboxybiphenyl-4-yl)methyl)-2-cyclopropyl-7-methyl-3H-imidazo(4,5-b)pyridine".mp OR "4'-((2-butyl-4-oxo-1,3-diazaspiro(4.4)non-1-en-3-yl)methyl)-N-(4,5-dimethyl-3-isoxazolyl)-2'-(ethoxymethyl)(1,1'-biphenyl)-2-sulfonamide".mp OR "57G709".mp OR "606A compound".mp OR "A 81988".mp OR "Abbott 81282".mp OR "Amlodipine Besylate, Olmesartan Medoxomil Drug Combination".mp OR "azilsartan medoxomil".mp OR "BMS 183920".mp OR "candesartan".mp OR "candesartan cilexetil".mp OR "dimethyl (1-methyl-1,3-benzimidazol-5-yl)aminomethylenepropanedioate".mp OR "enoltasosartan".mp OR "eprosartan".mp OR "GR 117289".mp OR "HN 65021".mp OR "Irbesartan".mp OR "KD3 671".mp OR "KR 31080".mp OR "KRH 594".mp OR "L 158809".mp OR "L 159913".mp OR "L 163017".mp OR "L 163082".mp OR "L 163958".mp OR "L 164282".mp OR "Losartan".mp OR "LR B-081".mp OR "N-(2-(6-((2-ethyl-5,7-dimethyl-3H-imidazo(4,5-b)pyridin-3-yl)methyl)quinolin-2-yl))trifluoromethanesulfonamide".mp OR "olmesartan".mp OR "Olmesartan Medoxomil".mp OR "PD 123319".mp OR "sacubitril-valsartan".mp OR "saprisartan potassium".mp OR "Saralasin".mp OR "SL 910102-90 DL".mp OR "tasosartan".mp OR "Telmisartan".mp OR "telmisartan amlodipine combination".mp OR "telmisartan, hydrochlorothiazide drug combination".mp OR "TH 142177".mp OR "UR 7247".mp OR "UR 7280".mp OR "Valsartan".mp OR "XR 510".mp OR "YM358".mp OR "ZD 7155".mp OR exp "nitric acid derivative"/ OR exp "Nitrates"/ OR "Nitrates".mp OR "Nitrate".mp OR "Tetranitrate".mp OR "Nicorandil".mp OR exp "serotonin uptake inhibitor"/ OR exp "Serotonin Uptake Inhibitors"/ OR "Serotonin Uptake Inhibitors".mp OR "Serotonin Uptake Inhibitor".mp OR "Serotonin re uptake Inhibitors".mp OR "Serotonin re uptake Inhibitor".mp OR "Serotonin reuptake Inhibitors".mp OR "Serotonin reuptake Inhibitor".mp OR "Selective serotonin reuptake inhibitors".mp OR "Selective serotonin reuptake inhibitor".mp OR "Selective serotonin re uptake inhibitors".mp OR "Selective serotonin re uptake inhibitor".mp OR "SSRI".mp OR "SSRIs".mp) NOT (conference review or conference abstract).pt)

**Web of Science**

<http://isiknowledge.com/wos>

TS=(("Scleroderma, Systemic" OR "systemic sclerosis" OR "Systemic Scleroderma" OR "SSc" OR "systemic sclerosis" OR "Diffuse Scleroderma" OR "Limited Scleroderma" OR "Scleroderma" OR "scleroderma*" OR "CREST syndrome") AND ("finger ulcer" OR "digital ulcers" OR "digital ulcer" OR "digital ulcer*" OR (("Fingers" OR "Figer" OR "Fingers" OR "Finger" OR "thumb" OR "Toes" OR "Toe" OR "toes" OR "toe" OR "hallux" OR "digital" OR "digit" OR "digits") AND ("Skin Ulcer" OR "Ulcer" OR "Ulcers" OR "ulcer*"))) AND ("Systemic therapy" OR "Systemic treatment" OR "Systemic treat*" OR "pharmacological treatment" OR "pharmacological treat*" OR "Systemic therapy" OR "Systemic therap*" OR "pharmacological therapy" OR "pharmacological therapy*" OR "Drug Therapy" OR "Drug Therapy" OR "Vasodilator Agents" OR "Vasodilator Agent" OR "Vasodilators" OR "Vasodilator" OR "Prostaglandin" OR "Prostaglandins" OR "Prostaglandins" OR "Prostaglandin" OR "Prostanoids" OR "Prostanoid" OR "Iloprost" OR "Iloprost" OR "Ciloprost" OR "Ventavis" OR "Epoprostenol" OR "Epoprostanol" OR "Prostaglandin I2" OR "Prostacyclin" OR "Veletri" OR "Epoprostenol Sodium" OR "Flolan" OR "treprostinil" OR "Treprostinil" OR "Orenitram" OR "Remodulin" OR "Phosphodiesterase V Inhibitor" OR "Phosphodiesterase 5 Inhibitors" OR "Phosphodiesterase 5 Inhibitors" OR "Phosphodiesterase 5 Inhibitor" OR "Sildenafil Citrate" OR "Sildenafil" OR "Revatio" OR "Homosildenafil" OR "Hydroxyhomosildenafil" OR "Viagra" OR "Acetildenafil" OR "Desmethylsildenafil" OR "Tadalafil" OR "Tadalafil" OR "Cialis" OR "Endothelin Receptor Antagonist" OR "Endothelin Receptor Antagonists" OR "Endothelin Receptor Antagonists" OR "Endothelin Receptor Antagonist" OR "Bosentan" OR "Bosentan" OR "Tracleer" OR "calcium channel blocking agent" OR "Calcium Channel Blockers" OR "Calcium Channel Blockers" OR "Calcium Channel Blocker" OR "Nifedipine" OR "Nifedipine" OR "Cordipin" OR "Cordipine" OR "Corinfar" OR "Korinfar" OR "Nifangin" OR "Procardia" OR "Vascard" OR "Adalat" OR "Fenigidin" OR "Amlodipine" OR "Amlodipine" OR "Amlodis" OR "Astudal" OR "Norvasc" OR "Istin" OR "Amlor" OR "Acetylcysteine" OR "Acetylcysteine" OR "N acetylcysteine" OR "N Acetyl L cysteine" OR "Mercapturic Acid" OR "Solmucol" OR "Genac" OR "Acemuc" OR "Acetabs" OR "NAC AL" OR "Acetylcystein" OR "Acetyst" OR "Airbron" OR "Alveolex" OR "Bromuc" OR "Azubronchin" OR "Bisolvon NAC" OR "Broncho Fips" OR "BronchoFips" OR "Broncholysin" OR "Broncoclar" OR "Codotussyl" OR "Cystamucil" OR "Dampo Mucopect" OR "Mucopect, Dampo" OR "durabronchal" OR "Larylin NAC" OR "Eurespiran" OR "Exomuc" OR "Fluimucil" OR "NAC Zambon" OR "Fabrol" OR "Fluprowit" OR "Optipect Hustengetränk" OR "Muco Sanigen" OR "Frekatuss" OR "Jenacystein" OR "Jenapharm" OR "Lantamed" OR "Lindocetyl" OR "M Pectil" OR "MPectil" OR "mentopin Acetylcystein" OR "Muciteran" OR "Mucomyst" OR "Acetylin" OR "Mucosil" OR "Mucosol" OR "Mucosolvin" OR "Siccoral" OR "Siran" OR "Ilube" OR "Hoestil" OR "acebraus" OR "Anti-platelet therapy" OR "antithrombocytic agent" OR "Platelet Aggregation Inhibitors" OR "Platelet Aggregation Inhibitors" OR "Platelet Aggregation Inhibitor" OR "Aspirin" OR "Aspirin" OR "Acetylsalicylic Acid" OR "Acylpyrin" OR "Aloxiprimum" OR "Colfarit" OR "Dispril" OR "Easprin" OR "Ecotrin" OR "Endosprin" OR "Magnecyl" OR "Micristin" OR "Polopirin" OR "Polopiryna" OR "Solprin" OR "Solupsan" OR "Zorprin" OR "Acetysal" OR "Dipyridamole" OR "Dipyridamole" OR "Cerebrovase" OR "Persantine" OR "Persantin" OR "Curantil" OR "Curantyl" OR "Kurantil" OR "Miosen" OR "Novo Dipiradol" OR "Antistenocardin" OR "Cléridium" OR "dipeptidyl carboxypeptidase inhibitor" OR "Angiotensin-Converting Enzyme Inhibitors" OR "Angiotensin-Converting Enzyme Inhibitors" OR "Angiotensin-Converting Enzyme Inhibitor" OR "ACE Inhibitors" OR "ACE Inhibitor" OR "(S)-malic acid 1'-O-beta-gentiobioside" OR "2-(2-(5-bromoindan-1-yl)-3-mercaptopropionylamino)-3-(1H-pyrrolo(2,3-b)pyridin-3-yl)propionic acid" OR "7-((2-(acetylthio)-1-oxo-3-methylpropyl)amino)-1,2,3,4,6,7,8,12b-octahydro-6-oxopyrido(2,1-a)(2)benzazepin-4-carboxyacid" OR "alacepril" OR "benazepril" OR "benazeprilat" OR "Captopril" OR "captopril, hydrochlorothiazide drug combination" OR "ceronapril" OR "Cilazapril" OR "cilazaprilat" OR "delapril" OR "Enalapril" OR "Enalaprilat" OR "Fosinopril" OR "fosinoprilat" OR "gemopatrilat" OR "glyceraldehyde 3-phosphate dehydrogenase (304-313)" OR "imidapril" OR "imidaprilat" OR "IRW peptide" OR "L-proline, N2-((1S)-1-carboxy-3-phenylpropyl)-N6-((4-hydroxyphenyl)iminomethyl)-L-lysyl-" OR "libenzapril" OR "Lisinopril" OR "LVV-hemorphin 6" OR "MDL 100240" OR "moexipril" OR "N-((1-((2-(acetylthio)-3-methyl-1-oxobutyl)amino)-1-cyclopentyl)carbonyl)-O-methyl-L-tyrosine ethyl ester" OR "N-(1-carboxy-3-phenylpropyl)-alanylalanine" OR "N-(2-(mercaptomethyl)-3-methylbutanoyl)-4-(1H-pyrazol-1-yl)phenylalanine" OR "omapatrilat" OR "Perindopril" OR "perindoprilat" OR "Quinapril" OR "quinaprilat" OR "Ramipril" OR "ramiprilat" OR "rentiapril" OR "sampatrilat" OR "spirapril" OR "temocapril hydrochloride" OR "Teprotide" OR "trandolapril" OR "valyl-prolyl-proline" OR "zofenopril" OR "angiotensin receptor antagonist" OR "Angiotensin Receptor Antagonists" OR "Angiotensin Receptor Antagonists" OR "Angiotensin Receptor Antagonist" OR "Angiotensin Receptor Blockers" OR "Angiotensin Receptor Blocker" OR "1-Sarcosine-8-Isoleucine Angiotensin II" OR "2-(butyryl-(2'-(4,5-dimethylisoxazol-3-ylsulfamoyl)biphenyl-4-ylmethyl)amino)-N-isopropyl-3-methylbutyramide" OR "2-(butyryl-(2'-(4-chloro-5-methylisoxazol-3-ylsulfamoyl)biphenyl-4-ylmethyl)amino)-N-isopropyl-3-methylbutyramide" OR "2-(butyryl-(2'-(4-fluoro-5-methylisoxazol-3-ylsulfamoyl)biphenyl-4-ylmethyl)amino)-N-isopropyl-3-methylbutyramide" OR "2-butyl-3-((2'-(1H-tetrazol-5-yl)(1,1'-biphenyl)-4-yl)methyl)-2,3-diazaspiro(4.4)nonane-1,4-dione" OR "2-methylsulfanyl-3-(2'-(2H-tetrazol-5-yl)biphenyl-4-ylmethyl)-3H-quinazolin-4-one" OR "3-((2'-(benzoylaminosulfonyl)(1,1'-biphenyl)-4-yl)methyl)-2-butyl-2,3-diazaspiro(4.4)nonane-1,4-dione" OR "3-((2'-carboxybiphenyl-4-yl)methyl)-2-cyclopropyl-7-methyl-3H-imidazo(4,5-b)pyridine" OR "4'-((2-butyl-4-oxo-1,3-diazaspiro(4.4)non-1-en-3-yl)methyl)-N-(4,5-dimethyl-3-isoxazolyl)-2'-(ethoxymethyl)(1,1'-biphenyl)-2-sulfonamide" OR "57G709" OR "606A compound" OR "A 81988" OR "Abbott 81282" OR "Amlodipine Besylate, Olmesartan Medoxomil Drug Combination" OR "azilsartan medoxomil" OR "BMS 183920" OR "candesartan" OR "candesartan cilexetil" OR "dimethyl (1-methyl-1,3-benzimidazol-5-yl)aminomethylenepropanedioate" OR "enoltasosartan" OR "eprosartan" OR "GR 117289" OR "HN 65021" OR "Irbesartan" OR "KD3 671" OR "KR 31080" OR "KRH 594" OR "L 158809" OR "L 159913" OR "L 163017" OR "L 163082" OR "L 163958" OR "L 164282" OR "Losartan" OR "LR B-081" OR "N-(2-(6-((2-ethyl-5,7-dimethyl-3H-imidazo(4,5-b)pyridin-3-yl)methyl)quinolin-2-yl))trifluoromethanesulfonamide" OR "olmesartan" OR "Olmesartan Medoxomil" OR "PD 123319" OR "sacubitril-valsartan" OR "saprisartan potassium" OR "Saralasin" OR "SL 910102-90 DL" OR "tasosartan" OR "Telmisartan" OR "telmisartan amlodipine combination" OR "telmisartan, hydrochlorothiazide drug combination" OR "TH 142177" OR "UR 7247" OR "UR 7280" OR "Valsartan" OR "XR 510" OR "YM358" OR "ZD 7155" OR "nitric acid derivative" OR "Nitrates" OR "Nitrates" OR "Nitrate" OR "Tetranitrate" OR "Nicorandil" OR "serotonin uptake inhibitor" OR "Serotonin Uptake Inhibitors" OR "Serotonin Uptake Inhibitors" OR "Serotonin Uptake Inhibitor" OR "Serotonin re uptake Inhibitors" OR "Serotonin re uptake Inhibitor" OR "Serotonin reuptake Inhibitors" OR "Serotonin reuptake Inhibitor" OR "Selective serotonin reuptake inhibitors" OR "Selective serotonin reuptake inhibitor" OR "Selective serotonin re uptake inhibitors" OR "Selective serotonin re uptake inhibitor" OR "SSRI" OR "SSRIs")) NOT DT=(meeting abstract)

**Cochrane**

<https://www.cochranelibrary.com/advanced-search/search-manager>

(("Scleroderma, Systemic" OR "systemic sclerosis" OR "Systemic Scleroderma" OR "SSc" OR "systemic sclerosis" OR "Diffuse Scleroderma" OR "Limited Scleroderma" OR "Scleroderma" OR "scleroderma*" OR "CREST syndrome") AND ("finger ulcer" OR "digital ulcers" OR "digital ulcer" OR "digital ulcer*" OR (("Fingers" OR "Figer" OR "Fingers" OR "Finger" OR "thumb" OR "Toes" OR "Toe" OR "toes" OR "toe" OR "hallux" OR "digital" OR "digit" OR "digits") AND ("Skin Ulcer" OR "Ulcer" OR "Ulcers" OR "ulcer*"))) AND ("Systemic therapy" OR "Systemic treatment" OR "Systemic treat*" OR "pharmacological treatment" OR "pharmacological treat*" OR "Systemic therapy" OR "Systemic therap*" OR "pharmacological therapy" OR "pharmacological therapy*" OR "Drug Therapy" OR "Drug Therapy" OR "Vasodilator Agents" OR "Vasodilator Agent" OR "Vasodilators" OR "Vasodilator" OR "Prostaglandin" OR "Prostaglandins" OR "Prostaglandins" OR "Prostaglandin" OR "Prostanoids" OR "Prostanoid" OR "Iloprost" OR "Iloprost" OR "Ciloprost" OR "Ventavis" OR "Epoprostenol" OR "Epoprostanol" OR "Prostaglandin I2" OR "Prostacyclin" OR "Veletri" OR "Epoprostenol Sodium" OR "Flolan" OR "treprostinil" OR "Treprostinil" OR "Orenitram" OR "Remodulin" OR "Phosphodiesterase V Inhibitor" OR "Phosphodiesterase 5 Inhibitors" OR "Phosphodiesterase 5 Inhibitors" OR "Phosphodiesterase 5 Inhibitor" OR "Sildenafil Citrate" OR "Sildenafil" OR "Revatio" OR "Homosildenafil" OR "Hydroxyhomosildenafil" OR "Viagra" OR "Acetildenafil" OR "Desmethylsildenafil" OR "Tadalafil" OR "Tadalafil" OR "Cialis" OR "Endothelin Receptor Antagonist" OR "Endothelin Receptor Antagonists" OR "Endothelin Receptor Antagonists" OR "Endothelin Receptor Antagonist" OR "Bosentan" OR "Bosentan" OR "Tracleer" OR "calcium channel blocking agent" OR "Calcium Channel Blockers" OR "Calcium Channel Blockers" OR "Calcium Channel Blocker" OR "Nifedipine" OR "Nifedipine" OR "Cordipin" OR "Cordipine" OR "Corinfar" OR "Korinfar" OR "Nifangin" OR "Procardia" OR "Vascard" OR "Adalat" OR "Fenigidin" OR "Amlodipine" OR "Amlodipine" OR "Amlodis" OR "Astudal" OR "Norvasc" OR "Istin" OR "Amlor" OR "Acetylcysteine" OR "Acetylcysteine" OR "N acetylcysteine" OR "N Acetyl L cysteine" OR "Mercapturic Acid" OR "Solmucol" OR "Genac" OR "Acemuc" OR "Acetabs" OR "NAC AL" OR "Acetylcystein" OR "Acetyst" OR "Airbron" OR "Alveolex" OR "Bromuc" OR "Azubronchin" OR "Bisolvon NAC" OR "Broncho Fips" OR "BronchoFips" OR "Broncholysin" OR "Broncoclar" OR "Codotussyl" OR "Cystamucil" OR "Dampo Mucopect" OR "Mucopect, Dampo" OR "durabronchal" OR "Larylin NAC" OR "Eurespiran" OR "Exomuc" OR "Fluimucil" OR "NAC Zambon" OR "Fabrol" OR "Fluprowit" OR "Optipect Hustengetränk" OR "Muco Sanigen" OR "Frekatuss" OR "Jenacystein" OR "Jenapharm" OR "Lantamed" OR "Lindocetyl" OR "M Pectil" OR "MPectil" OR "mentopin Acetylcystein" OR "Muciteran" OR "Mucomyst" OR "Acetylin" OR "Mucosil" OR "Mucosol" OR "Mucosolvin" OR "Siccoral" OR "Siran" OR "Ilube" OR "Hoestil" OR "acebraus" OR "Anti platelet therapy" OR "antithrombocytic agent" OR "Platelet Aggregation Inhibitors" OR "Platelet Aggregation Inhibitors" OR "Platelet Aggregation Inhibitor" OR "Aspirin" OR "Aspirin" OR "Acetylsalicylic Acid" OR "Acylpyrin" OR "Aloxiprimum" OR "Colfarit" OR "Dispril" OR "Easprin" OR "Ecotrin" OR "Endosprin" OR "Magnecyl" OR "Micristin" OR "Polopirin" OR "Polopiryna" OR "Solprin" OR "Solupsan" OR "Zorprin" OR "Acetysal" OR "Dipyridamole" OR "Dipyridamole" OR "Cerebrovase" OR "Persantine" OR "Persantin" OR "Curantil" OR "Curantyl" OR "Kurantil" OR "Miosen" OR "Novo Dipiradol" OR "Antistenocardin" OR "Cléridium" OR "dipeptidyl carboxypeptidase inhibitor" OR "Angiotensin Converting Enzyme Inhibitors" OR "Angiotensin Converting Enzyme Inhibitors" OR "Angiotensin Converting Enzyme Inhibitor" OR "ACE Inhibitors" OR "ACE Inhibitor" OR "(S) malic acid 1' O beta gentiobioside" OR "2 (2 (5 bromoindan 1 yl) 3 mercaptopropionylamino) 3 (1H pyrrolo(2,3 b)pyridin 3 yl)propionic acid" OR "7 ((2 (acetylthio) 1 oxo 3 methylpropyl)amino) 1,2,3,4,6,7,8,12b octahydro 6 oxopyrido(2,1 a)(2)benzazepin 4 carboxyacid" OR "alacepril" OR "benazepril" OR "benazeprilat" OR "Captopril" OR "captopril, hydrochlorothiazide drug combination" OR "ceronapril" OR "Cilazapril" OR "cilazaprilat" OR "delapril" OR "Enalapril" OR "Enalaprilat" OR "Fosinopril" OR "fosinoprilat" OR "gemopatrilat" OR "glyceraldehyde 3 phosphate dehydrogenase (304 313)" OR "imidapril" OR "imidaprilat" OR "IRW peptide" OR "L proline, N2 ((1S) 1 carboxy 3 phenylpropyl) N6 ((4 hydroxyphenyl)iminomethyl) L lysyl " OR "libenzapril" OR "Lisinopril" OR "LVV hemorphin 6" OR "MDL 100240" OR "moexipril" OR "N ((1 ((2 (acetylthio) 3 methyl 1 oxobutyl)amino) 1 cyclopentyl)carbonyl) O methyl L tyrosine ethyl ester" OR "N (1 carboxy 3 phenylpropyl) alanylalanine" OR "N (2 (mercaptomethyl) 3 methylbutanoyl) 4 (1H pyrazol 1 yl)phenylalanine" OR "omapatrilat" OR "Perindopril" OR "perindoprilat" OR "Quinapril" OR "quinaprilat" OR "Ramipril" OR "ramiprilat" OR "rentiapril" OR "sampatrilat" OR "spirapril" OR "temocapril hydrochloride" OR "Teprotide" OR "trandolapril" OR "valyl prolyl proline" OR "zofenopril" OR "angiotensin receptor antagonist" OR "Angiotensin Receptor Antagonists" OR "Angiotensin Receptor Antagonists" OR "Angiotensin Receptor Antagonist" OR "Angiotensin Receptor Blockers" OR "Angiotensin Receptor Blocker" OR "1 Sarcosine 8 Isoleucine Angiotensin II" OR "2 (butyryl (2' (4,5 dimethylisoxazol 3 ylsulfamoyl)biphenyl 4 ylmethyl)amino) N isopropyl 3 methylbutyramide" OR "2 (butyryl (2' (4 chloro 5 methylisoxazol 3 ylsulfamoyl)biphenyl 4 ylmethyl)amino) N isopropyl 3 methylbutyramide" OR "2 (butyryl (2' (4 fluoro 5 methylisoxazol 3 ylsulfamoyl)biphenyl 4 ylmethyl)amino) N isopropyl 3 methylbutyramide" OR "2 butyl 3 ((2' (1H tetrazol 5 yl)(1,1' biphenyl) 4 yl)methyl) 2,3 diazaspiro(4.4)nonane 1,4 dione" OR "2 methylsulfanyl 3 (2' (2H tetrazol 5 yl)biphenyl 4 ylmethyl) 3H quinazolin 4 one" OR "3 ((2' (benzoylaminosulfonyl)(1,1' biphenyl) 4 yl)methyl) 2 butyl 2,3 diazaspiro(4.4)nonane 1,4 dione" OR "3 ((2' carboxybiphenyl 4 yl)methyl) 2 cyclopropyl 7 methyl 3H imidazo(4,5 b)pyridine" OR "4' ((2 butyl 4 oxo 1,3 diazaspiro(4.4)non 1 en 3 yl)methyl) N (4,5 dimethyl 3 isoxazolyl) 2' (ethoxymethyl)(1,1' biphenyl) 2 sulfonamide" OR "57G709" OR "606A compound" OR "A 81988" OR "Abbott 81282" OR "Amlodipine Besylate, Olmesartan Medoxomil Drug Combination" OR "azilsartan medoxomil" OR "BMS 183920" OR "candesartan" OR "candesartan cilexetil" OR "dimethyl (1 methyl 1,3 benzimidazol 5 yl)aminomethylenepropanedioate" OR "enoltasosartan" OR "eprosartan" OR "GR 117289" OR "HN 65021" OR "Irbesartan" OR "KD3 671" OR "KR 31080" OR "KRH 594" OR "L 158809" OR "L 159913" OR "L 163017" OR "L 163082" OR "L 163958" OR "L 164282" OR "Losartan" OR "LR B 081" OR "N (2 (6 ((2 ethyl 5,7 dimethyl 3H imidazo(4,5 b)pyridin 3 yl)methyl)quinolin 2 yl))trifluoromethanesulfonamide" OR "olmesartan" OR "Olmesartan Medoxomil" OR "PD 123319" OR "sacubitril valsartan" OR "saprisartan potassium" OR "Saralasin" OR "SL 910102 90 DL" OR "tasosartan" OR "Telmisartan" OR "telmisartan amlodipine combination" OR "telmisartan, hydrochlorothiazide drug combination" OR "TH 142177" OR "UR 7247" OR "UR 7280" OR "Valsartan" OR "XR 510" OR "YM358" OR "ZD 7155" OR "nitric acid derivative" OR "Nitrates" OR "Nitrates" OR "Nitrate" OR "Tetranitrate" OR "Nicorandil" OR "serotonin uptake inhibitor" OR "Serotonin Uptake Inhibitors" OR "Serotonin Uptake Inhibitors" OR "Serotonin Uptake Inhibitor" OR "Serotonin re uptake Inhibitors" OR "Serotonin re uptake Inhibitor" OR "Serotonin reuptake Inhibitors" OR "Serotonin reuptake Inhibitor" OR "Selective serotonin reuptake inhibitors" OR "Selective serotonin reuptake inhibitor" OR "Selective serotonin re uptake inhibitors" OR "Selective serotonin re uptake inhibitor" OR "SSRI" OR "SSRIs")):ti,ab,kw NOT (conference abstract or meeting abstract):pt

**Emcare** <http://ovidsp.ovid.com/ovidweb.cgi?T=JS&NEWS=n&CSC=Y&PAGE=main&D=emcr>

((exp "Scleroderma, Systemic"/ OR exp "systemic sclerosis"/ OR "Systemic Scleroderma".mp OR "SSc".mp OR "systemic sclerosis".mp OR "Diffuse Scleroderma".mp OR "Limited Scleroderma".mp OR "Scleroderma".mp OR "scleroderma*".mp OR "CREST syndrome".mp) AND ("finger ulcer"/ OR "digital ulcers".mp OR "digital ulcer".mp OR "digital ulcer*".mp OR ((exp "Fingers"/ OR exp "Finger"/ OR "Fingers".mp OR "Finger".mp OR "thumb".mp OR exp "Toes"/ OR exp "Toe"/ OR "toes".mp OR "toe".mp OR "hallux".mp OR "digital".mp OR "digit".mp OR "digits".mp) AND (exp "Skin Ulcer"/ OR "Ulcer".mp OR "Ulcers".mp OR "ulcer*".mp))) AND (exp "Systemic therapy"/ OR "Systemic treatment".mp OR "Systemic treat*".mp OR "pharmacological treatment".mp OR "pharmacological treat*".mp OR "Systemic therapy".mp OR "Systemic therap*".mp OR "pharmacological therapy".mp OR "pharmacological therapy*".mp OR exp "Drug Therapy"/ OR "Drug Therapy".mp OR exp "Vasodilator Agents"/ OR exp "Vasodilator Agent"/ OR "Vasodilators".mp OR "Vasodilator".mp OR exp "Prostaglandin"/ OR exp "Prostaglandins"/ OR "Prostaglandins".mp OR "Prostaglandin".mp OR "Prostanoids".mp OR "Prostanoid".mp OR exp "Iloprost"/ OR "Iloprost".mp OR "Ciloprost".mp OR "Ventavis".mp OR exp "Epoprostenol"/ OR "Epoprostanol".mp OR "Prostaglandin I2".mp OR "Prostacyclin".mp OR "Veletri".mp OR "Epoprostenol Sodium".mp OR "Flolan".mp OR "treprostinil"/ OR "Treprostinil".mp OR "Orenitram".mp OR "Remodulin".mp OR exp "Phosphodiesterase V Inhibitor"/ OR exp "Phosphodiesterase 5 Inhibitors"/ OR "Phosphodiesterase 5 Inhibitors".mp OR "Phosphodiesterase 5 Inhibitor".mp OR exp "Sildenafil Citrate"/ OR "Sildenafil".mp OR "Revatio".mp OR "Homosildenafil".mp OR "Hydroxyhomosildenafil".mp OR "Viagra".mp OR "Acetildenafil".mp OR "Desmethylsildenafil".mp OR exp "Tadalafil"/ OR "Tadalafil".mp OR "Cialis".mp OR exp "Endothelin Receptor Antagonist"/ OR exp "Endothelin Receptor Antagonists"/ OR "Endothelin Receptor Antagonists".mp OR "Endothelin Receptor Antagonist".mp OR exp "Bosentan"/ OR "Bosentan".mp OR "Tracleer".mp OR exp "calcium channel blocking agent"/ OR exp "Calcium Channel Blockers"/ OR "Calcium Channel Blockers".mp OR "Calcium Channel Blocker".mp OR exp "Nifedipine"/ OR "Nifedipine".mp OR "Cordipin".mp OR "Cordipine".mp OR "Corinfar".mp OR "Korinfar".mp OR "Nifangin".mp OR "Procardia".mp OR "Vascard".mp OR "Adalat".mp OR "Fenigidin".mp OR exp "Amlodipine"/ OR "Amlodipine".mp OR "Amlodis".mp OR "Astudal".mp OR "Norvasc".mp OR "Istin".mp OR "Amlor".mp OR exp "Acetylcysteine"/ OR "Acetylcysteine".mp OR "N acetylcysteine".mp OR "N Acetyl L cysteine".mp OR "Mercapturic Acid".mp OR "Solmucol".mp OR "Genac".mp OR "Acemuc".mp OR "Acetabs".mp OR "NAC AL".mp OR "Acetylcystein".mp OR "Acetyst".mp OR "Airbron".mp OR "Alveolex".mp OR "Bromuc".mp OR "Azubronchin".mp OR "Bisolvon NAC".mp OR "Broncho Fips".mp OR "BronchoFips".mp OR "Broncholysin".mp OR "Broncoclar".mp OR "Codotussyl".mp OR "Cystamucil".mp OR "Dampo Mucopect".mp OR "Mucopect, Dampo".mp OR "durabronchal".mp OR "Larylin NAC".mp OR "Eurespiran".mp OR "Exomuc".mp OR "Fluimucil".mp OR "NAC Zambon".mp OR "Fabrol".mp OR "Fluprowit".mp OR "Optipect Hustengetränk".mp OR "Muco Sanigen".mp OR "Frekatuss".mp OR "Jenacystein".mp OR "Jenapharm".mp OR "Lantamed".mp OR "Lindocetyl".mp OR "M Pectil".mp OR "MPectil".mp OR "mentopin Acetylcystein".mp OR "Muciteran".mp OR "Mucomyst".mp OR "Acetylin".mp OR "Mucosil".mp OR "Mucosol".mp OR "Mucosolvin".mp OR "Siccoral".mp OR "Siran".mp OR "Ilube".mp OR "Hoestil".mp OR "acebraus".mp OR "Anti-platelet therapy".mp OR exp "antithrombocytic agent"/ OR exp "Platelet Aggregation Inhibitors"/ OR "Platelet Aggregation Inhibitors".mp OR "Platelet Aggregation Inhibitor".mp OR "Aspirin"/ OR "Aspirin".mp OR "Acetylsalicylic Acid".mp OR "Acylpyrin".mp OR "Aloxiprimum".mp OR "Colfarit".mp OR "Dispril".mp OR "Easprin".mp OR "Ecotrin".mp OR "Endosprin".mp OR "Magnecyl".mp OR "Micristin".mp OR "Polopirin".mp OR "Polopiryna".mp OR "Solprin".mp OR "Solupsan".mp OR "Zorprin".mp OR "Acetysal".mp OR exp "Dipyridamole"/ OR "Dipyridamole".mp OR "Cerebrovase".mp OR "Persantine".mp OR "Persantin".mp OR "Curantil".mp OR "Curantyl".mp OR "Kurantil".mp OR "Miosen".mp OR "Novo Dipiradol".mp OR "Antistenocardin".mp OR "Cléridium".mp OR exp "dipeptidyl carboxypeptidase inhibitor"/ OR exp "Angiotensin-Converting Enzyme Inhibitors"/ OR "Angiotensin-Converting Enzyme Inhibitors".mp OR "Angiotensin-Converting Enzyme Inhibitor".mp OR "ACE Inhibitors".mp OR "ACE Inhibitor".mp OR "(S)-malic acid 1'-O-beta-gentiobioside".mp OR "2-(2-(5-bromoindan-1-yl)-3-mercaptopropionylamino)-3-(1H-pyrrolo(2,3-b)pyridin-3-yl)propionic acid".mp OR "7-((2-(acetylthio)-1-oxo-3-methylpropyl)amino)-1,2,3,4,6,7,8,12b-octahydro-6-oxopyrido(2,1-a)(2)benzazepin-4-carboxyacid".mp OR "alacepril".mp OR "benazepril".mp OR "benazeprilat".mp OR "Captopril".mp OR "captopril, hydrochlorothiazide drug combination".mp OR "ceronapril".mp OR "Cilazapril".mp OR "cilazaprilat".mp OR "delapril".mp OR "Enalapril".mp OR "Enalaprilat".mp OR "Fosinopril".mp OR "fosinoprilat".mp OR "gemopatrilat".mp OR "glyceraldehyde 3-phosphate dehydrogenase (304-313)".mp OR "imidapril".mp OR "imidaprilat".mp OR "IRW peptide".mp OR "L-proline, N2-((1S)-1-carboxy-3-phenylpropyl)-N6-((4-hydroxyphenyl)iminomethyl)-L-lysyl-".mp OR "libenzapril".mp OR "Lisinopril".mp OR "LVV-hemorphin 6".mp OR "MDL 100240".mp OR "moexipril".mp OR "N-((1-((2-(acetylthio)-3-methyl-1-oxobutyl)amino)-1-cyclopentyl)carbonyl)-O-methyl-L-tyrosine ethyl ester".mp OR "N-(1-carboxy-3-phenylpropyl)-alanylalanine".mp OR "N-(2-(mercaptomethyl)-3-methylbutanoyl)-4-(1H-pyrazol-1-yl)phenylalanine".mp OR "omapatrilat".mp OR "Perindopril".mp OR "perindoprilat".mp OR "Quinapril".mp OR "quinaprilat".mp OR "Ramipril".mp OR "ramiprilat".mp OR "rentiapril".mp OR "sampatrilat".mp OR "spirapril".mp OR "temocapril hydrochloride".mp OR "Teprotide".mp OR "trandolapril".mp OR "valyl-prolyl-proline".mp OR "zofenopril".mp OR exp "angiotensin receptor antagonist"/ OR exp "Angiotensin Receptor Antagonists"/ OR "Angiotensin Receptor Antagonists".mp OR "Angiotensin Receptor Antagonist".mp OR "Angiotensin Receptor Blockers".mp OR "Angiotensin Receptor Blocker".mp OR "1-Sarcosine-8-Isoleucine Angiotensin II".mp OR "2-(butyryl-(2'-(4,5-dimethylisoxazol-3-ylsulfamoyl)biphenyl-4-ylmethyl)amino)-N-isopropyl-3-methylbutyramide".mp OR "2-(butyryl-(2'-(4-chloro-5-methylisoxazol-3-ylsulfamoyl)biphenyl-4-ylmethyl)amino)-N-isopropyl-3-methylbutyramide".mp OR "2-(butyryl-(2'-(4-fluoro-5-methylisoxazol-3-ylsulfamoyl)biphenyl-4-ylmethyl)amino)-N-isopropyl-3-methylbutyramide".mp OR "2-butyl-3-((2'-(1H-tetrazol-5-yl)(1,1'-biphenyl)-4-yl)methyl)-2,3-diazaspiro(4.4)nonane-1,4-dione".mp OR "2-methylsulfanyl-3-(2'-(2H-tetrazol-5-yl)biphenyl-4-ylmethyl)-3H-quinazolin-4-one".mp OR "3-((2'-(benzoylaminosulfonyl)(1,1'-biphenyl)-4-yl)methyl)-2-butyl-2,3-diazaspiro(4.4)nonane-1,4-dione".mp OR "3-((2'-carboxybiphenyl-4-yl)methyl)-2-cyclopropyl-7-methyl-3H-imidazo(4,5-b)pyridine".mp OR "4'-((2-butyl-4-oxo-1,3-diazaspiro(4.4)non-1-en-3-yl)methyl)-N-(4,5-dimethyl-3-isoxazolyl)-2'-(ethoxymethyl)(1,1'-biphenyl)-2-sulfonamide".mp OR "57G709".mp OR "606A compound".mp OR "A 81988".mp OR "Abbott 81282".mp OR "Amlodipine Besylate, Olmesartan Medoxomil Drug Combination".mp OR "azilsartan medoxomil".mp OR "BMS 183920".mp OR "candesartan".mp OR "candesartan cilexetil".mp OR "dimethyl (1-methyl-1,3-benzimidazol-5-yl)aminomethylenepropanedioate".mp OR "enoltasosartan".mp OR "eprosartan".mp OR "GR 117289".mp OR "HN 65021".mp OR "Irbesartan".mp OR "KD3 671".mp OR "KR 31080".mp OR "KRH 594".mp OR "L 158809".mp OR "L 159913".mp OR "L 163017".mp OR "L 163082".mp OR "L 163958".mp OR "L 164282".mp OR "Losartan".mp OR "LR B-081".mp OR "N-(2-(6-((2-ethyl-5,7-dimethyl-3H-imidazo(4,5-b)pyridin-3-yl)methyl)quinolin-2-yl))trifluoromethanesulfonamide".mp OR "olmesartan".mp OR "Olmesartan Medoxomil".mp OR "PD 123319".mp OR "sacubitril-valsartan".mp OR "saprisartan potassium".mp OR "Saralasin".mp OR "SL 910102-90 DL".mp OR "tasosartan".mp OR "Telmisartan".mp OR "telmisartan amlodipine combination".mp OR "telmisartan, hydrochlorothiazide drug combination".mp OR "TH 142177".mp OR "UR 7247".mp OR "UR 7280".mp OR "Valsartan".mp OR "XR 510".mp OR "YM358".mp OR "ZD 7155".mp OR exp "nitric acid derivative"/ OR exp "Nitrates"/ OR "Nitrates".mp OR "Nitrate".mp OR "Tetranitrate".mp OR "Nicorandil".mp OR exp "serotonin uptake inhibitor"/ OR exp "Serotonin Uptake Inhibitors"/ OR "Serotonin Uptake Inhibitors".mp OR "Serotonin Uptake Inhibitor".mp OR "Serotonin re uptake Inhibitors".mp OR "Serotonin re uptake Inhibitor".mp OR "Serotonin reuptake Inhibitors".mp OR "Serotonin reuptake Inhibitor".mp OR "Selective serotonin reuptake inhibitors".mp OR "Selective serotonin reuptake inhibitor".mp OR "Selective serotonin re uptake inhibitors".mp OR "Selective serotonin re uptake inhibitor".mp OR "SSRI".mp OR "SSRIs".mp))

**Academic Search Premier**

<http://search.ebscohost.com/login.aspx?authtype=ip,uid&profile=lumc&defaultdb=aph>

(TI("Scleroderma, Systemic" OR "systemic sclerosis" OR "Systemic Scleroderma" OR "SSc" OR "systemic sclerosis" OR "Diffuse Scleroderma" OR "Limited Scleroderma" OR "Scleroderma" OR "scleroderma*" OR "CREST syndrome") AND TI("finger ulcer" OR "digital ulcers" OR "digital ulcer" OR "digital ulcer*" OR (("Fingers" OR "Figer" OR "Fingers" OR "Finger" OR "thumb" OR "Toes" OR "Toe" OR "toes" OR "toe" OR "hallux" OR "digital" OR "digit" OR "digits") AND ("Skin Ulcer" OR "Ulcer" OR "Ulcers" OR "ulcer*"))) AND TX("Systemic therapy" OR "Systemic treatment" OR "Systemic treat*" OR "pharmacological treatment" OR "pharmacological treat*" OR "Systemic therapy" OR "Systemic therap*" OR "pharmacological therapy" OR "pharmacological therapy*" OR "Drug Therapy" OR "Drug Therapy" OR "Vasodilator Agents" OR "Vasodilator Agent" OR "Vasodilators" OR "Vasodilator" OR "Prostaglandin" OR "Prostaglandins" OR "Prostaglandins" OR "Prostaglandin" OR "Prostanoids" OR "Prostanoid" OR "Iloprost" OR "Iloprost" OR "Ciloprost" OR "Ventavis" OR "Epoprostenol" OR "Epoprostanol" OR "Prostaglandin I2" OR "Prostacyclin" OR "Veletri" OR "Epoprostenol Sodium" OR "Flolan" OR "treprostinil" OR "Treprostinil" OR "Orenitram" OR "Remodulin" OR "Phosphodiesterase V Inhibitor" OR "Phosphodiesterase 5 Inhibitors" OR "Phosphodiesterase 5 Inhibitors" OR "Phosphodiesterase 5 Inhibitor" OR "Sildenafil Citrate" OR "Sildenafil" OR "Revatio" OR "Homosildenafil" OR "Hydroxyhomosildenafil" OR "Viagra" OR "Acetildenafil" OR "Desmethylsildenafil" OR "Tadalafil" OR "Tadalafil" OR "Cialis" OR "Endothelin Receptor Antagonist" OR "Endothelin Receptor Antagonists" OR "Endothelin Receptor Antagonists" OR "Endothelin Receptor Antagonist" OR "Bosentan" OR "Bosentan" OR "Tracleer" OR "calcium channel blocking agent" OR "Calcium Channel Blockers" OR "Calcium Channel Blockers" OR "Calcium Channel Blocker" OR "Nifedipine" OR "Nifedipine" OR "Cordipin" OR "Cordipine" OR "Corinfar" OR "Korinfar" OR "Nifangin" OR "Procardia" OR "Vascard" OR "Adalat" OR "Fenigidin" OR "Amlodipine" OR "Amlodipine" OR "Amlodis" OR "Astudal" OR "Norvasc" OR "Istin" OR "Amlor" OR "Acetylcysteine" OR "Acetylcysteine" OR "N acetylcysteine" OR "N Acetyl L cysteine" OR "Mercapturic Acid" OR "Solmucol" OR "Genac" OR "Acemuc" OR "Acetabs" OR "NAC AL" OR "Acetylcystein" OR "Acetyst" OR "Airbron" OR "Alveolex" OR "Bromuc" OR "Azubronchin" OR "Bisolvon NAC" OR "Broncho Fips" OR "BronchoFips" OR "Broncholysin" OR "Broncoclar" OR "Codotussyl" OR "Cystamucil" OR "Dampo Mucopect" OR "Mucopect, Dampo" OR "durabronchal" OR "Larylin NAC" OR "Eurespiran" OR "Exomuc" OR "Fluimucil" OR "NAC Zambon" OR "Fabrol" OR "Fluprowit" OR "Optipect Hustengetränk" OR "Muco Sanigen" OR "Frekatuss" OR "Jenacystein" OR "Jenapharm" OR "Lantamed" OR "Lindocetyl" OR "M Pectil" OR "MPectil" OR "mentopin Acetylcystein" OR "Muciteran" OR "Mucomyst" OR "Acetylin" OR "Mucosil" OR "Mucosol" OR "Mucosolvin" OR "Siccoral" OR "Siran" OR "Ilube" OR "Hoestil" OR "acebraus" OR "Anti platelet therapy" OR "antithrombocytic agent" OR "Platelet Aggregation Inhibitors" OR "Platelet Aggregation Inhibitors" OR "Platelet Aggregation Inhibitor" OR "Aspirin" OR "Aspirin" OR "Acetylsalicylic Acid" OR "Acylpyrin" OR "Aloxiprimum" OR "Colfarit" OR "Dispril" OR "Easprin" OR "Ecotrin" OR "Endosprin" OR "Magnecyl" OR "Micristin" OR "Polopirin" OR "Polopiryna" OR "Solprin" OR "Solupsan" OR "Zorprin" OR "Acetysal" OR "Dipyridamole" OR "Dipyridamole" OR "Cerebrovase" OR "Persantine" OR "Persantin" OR "Curantil" OR "Curantyl" OR "Kurantil" OR "Miosen" OR "Novo Dipiradol" OR "Antistenocardin" OR "Cléridium" OR "dipeptidyl carboxypeptidase inhibitor" OR "Angiotensin Converting Enzyme Inhibitors" OR "Angiotensin Converting Enzyme Inhibitors" OR "Angiotensin Converting Enzyme Inhibitor" OR "ACE Inhibitors" OR "ACE Inhibitor" OR "(S) malic acid 1' O beta gentiobioside" OR "2 (2 (5 bromoindan 1 yl) 3 mercaptopropionylamino) 3 (1H pyrrolo(2,3 b)pyridin 3 yl)propionic acid" OR "7 ((2 (acetylthio) 1 oxo 3 methylpropyl)amino) 1,2,3,4,6,7,8,12b octahydro 6 oxopyrido(2,1 a)(2)benzazepin 4 carboxyacid" OR "alacepril" OR "benazepril" OR "benazeprilat" OR "Captopril" OR "captopril, hydrochlorothiazide drug combination" OR "ceronapril" OR "Cilazapril" OR "cilazaprilat" OR "delapril" OR "Enalapril" OR "Enalaprilat" OR "Fosinopril" OR "fosinoprilat" OR "gemopatrilat" OR "glyceraldehyde 3 phosphate dehydrogenase (304 313)" OR "imidapril" OR "imidaprilat" OR "IRW peptide" OR "L proline, N2 ((1S) 1 carboxy 3 phenylpropyl) N6 ((4 hydroxyphenyl)iminomethyl) L lysyl " OR "libenzapril" OR "Lisinopril" OR "LVV hemorphin 6" OR "MDL 100240" OR "moexipril" OR "N ((1 ((2 (acetylthio) 3 methyl 1 oxobutyl)amino) 1 cyclopentyl)carbonyl) O methyl L tyrosine ethyl ester" OR "N (1 carboxy 3 phenylpropyl) alanylalanine" OR "N (2 (mercaptomethyl) 3 methylbutanoyl) 4 (1H pyrazol 1 yl)phenylalanine" OR "omapatrilat" OR "Perindopril" OR "perindoprilat" OR "Quinapril" OR "quinaprilat" OR "Ramipril" OR "ramiprilat" OR "rentiapril" OR "sampatrilat" OR "spirapril" OR "temocapril hydrochloride" OR "Teprotide" OR "trandolapril" OR "valyl prolyl proline" OR "zofenopril" OR "angiotensin receptor antagonist" OR "Angiotensin Receptor Antagonists" OR "Angiotensin Receptor Antagonists" OR "Angiotensin Receptor Antagonist" OR "Angiotensin Receptor Blockers" OR "Angiotensin Receptor Blocker" OR "1 Sarcosine 8 Isoleucine Angiotensin II" OR "2 (butyryl (2' (4,5 dimethylisoxazol 3 ylsulfamoyl)biphenyl 4 ylmethyl)amino) N isopropyl 3 methylbutyramide" OR "2 (butyryl (2' (4 chloro 5 methylisoxazol 3 ylsulfamoyl)biphenyl 4 ylmethyl)amino) N isopropyl 3 methylbutyramide" OR "2 (butyryl (2' (4 fluoro 5 methylisoxazol 3 ylsulfamoyl)biphenyl 4 ylmethyl)amino) N isopropyl 3 methylbutyramide" OR "2 butyl 3 ((2' (1H tetrazol 5 yl)(1,1' biphenyl) 4 yl)methyl) 2,3 diazaspiro(4.4)nonane 1,4 dione" OR "2 methylsulfanyl 3 (2' (2H tetrazol 5 yl)biphenyl 4 ylmethyl) 3H quinazolin 4 one" OR "3 ((2' (benzoylaminosulfonyl)(1,1' biphenyl) 4 yl)methyl) 2 butyl 2,3 diazaspiro(4.4)nonane 1,4 dione" OR "3 ((2' carboxybiphenyl 4 yl)methyl) 2 cyclopropyl 7 methyl 3H imidazo(4,5 b)pyridine" OR "4' ((2 butyl 4 oxo 1,3 diazaspiro(4.4)non 1 en 3 yl)methyl) N (4,5 dimethyl 3 isoxazolyl) 2' (ethoxymethyl)(1,1' biphenyl) 2 sulfonamide" OR "57G709" OR "606A compound" OR "A 81988" OR "Abbott 81282" OR "Amlodipine Besylate, Olmesartan Medoxomil Drug Combination" OR "azilsartan medoxomil" OR "BMS 183920" OR "candesartan" OR "candesartan cilexetil" OR "dimethyl (1 methyl 1,3 benzimidazol 5 yl)aminomethylenepropanedioate" OR "enoltasosartan" OR "eprosartan" OR "GR 117289" OR "HN 65021" OR "Irbesartan" OR "KD3 671" OR "KR 31080" OR "KRH 594" OR "L 158809" OR "L 159913" OR "L 163017" OR "L 163082" OR "L 163958" OR "L 164282" OR "Losartan" OR "LR B 081" OR "N (2 (6 ((2 ethyl 5,7 dimethyl 3H imidazo(4,5 b)pyridin 3 yl)methyl)quinolin 2 yl))trifluoromethanesulfonamide" OR "olmesartan" OR "Olmesartan Medoxomil" OR "PD 123319" OR "sacubitril valsartan" OR "saprisartan potassium" OR "Saralasin" OR "SL 910102 90 DL" OR "tasosartan" OR "Telmisartan" OR "telmisartan amlodipine combination" OR "telmisartan, hydrochlorothiazide drug combination" OR "TH 142177" OR "UR 7247" OR "UR 7280" OR "Valsartan" OR "XR 510" OR "YM358" OR "ZD 7155" OR "nitric acid derivative" OR "Nitrates" OR "Nitrates" OR "Nitrate" OR "Tetranitrate" OR "Nicorandil" OR "serotonin uptake inhibitor" OR "Serotonin Uptake Inhibitors" OR "Serotonin Uptake Inhibitors" OR "Serotonin Uptake Inhibitor" OR "Serotonin re uptake Inhibitors" OR "Serotonin re uptake Inhibitor" OR "Serotonin reuptake Inhibitors" OR "Serotonin reuptake Inhibitor" OR "Selective serotonin reuptake inhibitors" OR "Selective serotonin reuptake inhibitor" OR "Selective serotonin re uptake inhibitors" OR "Selective serotonin re uptake inhibitor" OR "SSRI" OR "SSRIs"))

**Supplementary Data S3: Studies excluded at full text review**

**Wrong outcome (n=42)**

1. Narvaez, J., et al. (2019). "Effectiveness and safety of rituximab for the treatment of refractory systemic sclerosis associated calcinosis: A case series and systematic review of the literature." *Autoimmun Rev* 18(3): 262-269.
2. Taniguchi, T., et al. (2012). "Effects of bosentan on nondigital ulcers in patients with systemic sclerosis." *Br J Dermatol* 166(2): 417-421.
3. Valenzuela, A., et al. (2020). "Calcinosis is associated with ischemic manifestations and increased disability in patients with systemic sclerosis." *Semin Arthritis Rheum* 50(5): 891-896.
4. Martin, M. F. and J. E. Tooke (1982). "Effects of prostaglandin E1 on microvascular haemodynamics in progressive systemic sclerosis." *Br Med J* 285(6356): 1688-1690.
5. Bali, G., et al. (2011). "Discontinuing long-term Iloprost treatment for Raynaud's Phenomenon and systemic sclerosis: a single-center, randomized, placebo-controlled, double-blind study." *Acta Dermatovenerol Alp Pannonica Adriat* 20(1): 13-21.
6. Altomare, G. F., et al. (1988). "Ketanserin in the treatment of progressive systemic sclerosis." *Angiology* 39(7 Pt 1): 583-586.
7. Boonstra, M., et al. (2017). "Rituximab in early systemic sclerosis." *RMD Open* 3(2).
8. Bose, N., et al. (2015). "Evaluation of the effect of ambrisentan on digital microvascular flow in patients with systemic sclerosis using laser Doppler perfusion imaging: a 12-week randomized double-blind placebo controlled trial." *Arthritis Res Ther* 17: 10.
9. Zachariae, H., et al. (1990). "Cyclosporin A treatment of systemic sclerosis." *Br J Dermatol* 122(5): 677-681.
10. Wigley, F. M., et al. (1998). "Oral iloprost treatment in patients with Raynaud's phenomenon secondary to systemic sclerosis: a multicenter, placebo-controlled, double-blind study." *Arthritis Rheum* 41(4): 670-677.
11. Sumida, H., et al. (2016). "Effect of ambrisentan on peripheral circulation in patients with systemic sclerosis." *Mod Rheumatol* 26(3): 454-457.
12. Murdaca, G., et al. (2016). "Beneficial effects of long-term treatment with bosentan on the development of pulmonary arterial hypertension in patients with systemic sclerosis." *J Int Med Res* 44(Suppl 1): 85-89.
13. Rezus, E., et al. (2020). "A patient-centered approach to the burden of symptoms in patients with scleroderma treated with Bosentan: A prospective single-center observational study." *Exp Ther Med* 19(3): 1739-1746.
14. Shah, A. A., et al. (2013). "Open label study of escalating doses of oral treprostinil diethanolamine in patients with systemic sclerosis and digital ischemia: pharmacokinetics and correlation with digital perfusion." *Arthritis Res Ther* 15(2): R54.
15. Negrini, S., et al. (2019). "Iloprost use and medical management of systemic sclerosis-related vasculopathy in Italian tertiary referral centers: results from the PROSIT study." *Clin Exp Med* 19(3): 357-366.
16. Nguyen, V. A., et al. (2010). "Effect of the dual endothelin receptor antagonist bosentan on Raynaud's phenomenon secondary to systemic sclerosis: a double-blind prospective, randomized, placebo-controlled pilot study." *Rheumatology (Oxford)* 49(3): 583-587.
17. Parisi, S., et al. (2014). "Efficacy of bosentan in the treatment of Raynaud's phenomenon in patients with systemic sclerosis never treated with prostanoids." *Reumatismo* 65(6): 286-291.
18. Rosato, E., et al. (2010). "Bosentan improves skin perfusion of hands in patients with systemic sclerosis with pulmonary arterial hypertension." *J Rheumatol* 37(12): 2531-2539.
19. Schioppo, T., et al. (2018). "Acute and chronic effects of two different intravenous iloprost regimens in systemic sclerosis: a pragmatic non-randomized trial." *Rheumatology (Oxford)* 57(8): 1408-1416.
20. Martin, M. F., et al. (1981). "Prostaglandin E1 infusions for vascular insufficiency in progressive systemic sclerosis." *Ann Rheum Dis* 40(4): 350-354.
21. Romaniello, A., et al. (2014). "In systemic sclerosis patients, bosentan is safe and effective for digital ulcer prevention and it seems to attenuate the development of pulmonary arterial hypertension." *Rheumatology (Oxford)* 53(3): 570-571.
22. Uslu, U., et al. (2018). "Systemic therapy with calcitonin has positive clinical effects on systemic sclerosis in patients with cutaneous manifestations." *Eur J Dermatol* 28(3): 364-369.
23. Meijs, J., et al. (2015). “Blood flow in the hands of a predefined homogeneous systemic sclerosis population: the presence of digital ulcers and the improvement with bosentan.” *Rheumatology (Oxford)* 54(2) 262-269.
24. Rotondo, C., et al. (2018). “Evidence for increase in finger blood flow, evaluated by laser Doppler flowmetry, following iloprost infusion in patients with systemic sclerosis: a week-long observational longitudinal study.” *Scand J Rheumatol* 47(4) 311-318.
25. Ferri, C., et al. (1987). "Plasma exchange in the treatment of progressive systemic sclerosis." *Plasma Ther Transfus Technol* 8(2): 169-176.
26. O’Reilly, M. J. G., et al. (1979). "Plasma exchange and Raynaud’s phenomenon - its asssessment by Doppler ultrasound velocimetry." *Br J Surg* 66(10): 712-715.
27. Dau, P. C. and J. P. Callahan (1994). "Immune modulation during treatment of systemic sclerosis with plasmapheresis and immunosuppressive drugs." *Clin Immunol Immunopathol* 70(2): 159-165.
28. Pourrat, J. P., et al. (1987). "Plasma exchange therapy in progressive systemic sclerosis." *Plasma Ther Transfus Technol* 8(2): 113-118.
29. Lukac, J., et al. (1985). "Effect of ketanserin on Raynaud's phenomenon in progressive systemic sclerosis: a double-blind trial." *Drugs Exp Clin Res* 11(9): 659-663.
30. Lukac, J., et al. (1991). "Long-term ketanserin treatment in patients with systemic sclerosis and Raynaud's phenomenon." *Curr Therapeutic Res* 50(6): 869-877.
31. Gliddon, A. E., et al. (2007). "Prevention of vascular damage in scleroderma and autoimmune Raynaud's phenomenon: a multicenter, randomized, double-blind, placebo-controlled trial of the angiotensin-converting enzyme inhibitor quinapril." *Arthritis Rheum* 56(11): 3837-3846.
32. Black, C., et al. (1998). "Oral iloprost in Raynaud’s phenomenon secondary to systemic sclerosis: A multicentre, placebo-controlled, dose-comparison study." *Br J Rheumatol* 37: 952-960.
33. Baron, M., et al. (2016). "Calcinosis is associated with digital ischaemia in systemic sclerosis-a longitudinal study." *Rheumatology (Oxford)* 55(12): 2148-2155.
34. Souza, E. J. R., et al. (2017). "Geographic variation as a risk factor for digital ulcers in systemic sclerosis patients: a multicentre registry." *Scand J Rheumatol* 46(4): 288-295.
35. Herrgott, I., et al. (2008). "Management of cutaneous vascular complications in systemic scleroderma: Experience from the German network." *Rheumatol Int* 28(10): 1023-1029.
36. Giuggioli, D., et al. (2018). "Scleroderma skin ulcers definition, classification and treatment strategies our experience and review of the literature." *Autoimmun Rev* 17(2): 155-164.
37. Caramaschi, P., et al. (2012). "Digital amputation in systemic sclerosis: prevalence and clinical associations. A retrospective longitudinal study." *J Rheumatol* 39(8): 1648-1653.
38. Zhang, H., et al. (2017). “Sustained benefit from combined plasmapheresis and allogenic mesenchymal stem cells transplantation therapy in systemic sclerosis.” *Arthritis Res Ther* 19(1):165
39. Keyszer, G. et al. (2011). “Treatment of severe progressive systemic sclerosis with transplantation of mesenchymal stromal cells from allogeneic related donors: report of five cases.” *Arthritis Rheum* 63(8): 2540-2.
40. Escobar-Soto, et al. (2021). “Human mesenchymal stem cells for the management of systemic sclerosis. Systematic review.” *Autoimmun Rev* 20(6): 102831
41. Khanna, D. et al. (2022). “Long-term safety and efficacy of tocilizumab in early systemic-sclerosis-interstitial lung disease: Open-label extension of a phase 3 randomized controlled trial.” *Am J Respir Crit Care Med* 205(6): 674-84.
42. Panopoulos, S.T. (2022). “Anti-interleukin 6 therapy effect for refractory joint and skin involvement in systemic sclerosis: A real-world, single-center experience.” *J Rheumatol* 49(1): 68-73.

**Wrong study design (n=30)**

1. Blagojevic, J., et al. (2020). "Use of vasoactive/vasodilating drugs for systemic sclerosis (SSc)-related digital ulcers (DUs) in expert tertiary centres: results from the analysis of the observational real-life DeSScipher study." *Clin Rheumatol* 39(1): 27-36.
2. Gore, J. and R. Silver (2005). "Oral sildenafil for the treatment of Raynaud's phenomenon and digital ulcers secondary to systemic sclerosis." *Ann Rheum Dis* 64(9): 1387.
3. Hachulla, E., et al. (2007). "Natural history of ischaemic digital ulcers in systemic sclerosis: single-center retrospective longitudinal study." *J Rheumatol* 34(12): 2423-2430.
4. Agard, C., et al. (2014). "Use of bosentan for digital ulcers related to systemic sclerosis: a real-life retrospective French study of 89 patients treated since specific approval." *Scand J Rheumatol* 43(5): 398-402.
5. Nihtyanova, S. I., et al. (2008). "Clinical burden of digital vasculopathy in limited and diffuse cutaneous systemic sclerosis." *Ann Rheum Dis* 67(1): 120-123.
6. Souza, E. J. R., et al. (2017). "Geographic variation as a risk factor for digital ulcers in systemic sclerosis patients: a multicentre registry." *Scand J Rheumatol* 46(4): 288-295.
7. Matucci-Cerinic, M., et al. (2016). "Elucidating the burden of recurrent and chronic digital ulcers in systemic sclerosis: long-term results from the DUO Registry." *Ann Rheum Dis* 75(10): 1770-1776.
8. Caramaschi, P., et al. (2012). "Severe vascular complications in patients aVected by systemic sclerosis cyclically treated with iloprost." *Rheumatol Int* 32(7): 1933-1938.
9. De Cata, A., et al. (2016). "Digital ulcers in scleroderma patients: A retrospective observational study." *Int J Immunopathol Pharmacol* 29(2): 180-187.
10. Foti, R., et al. (2017). "Long-term clinical stabilization of scleroderma patients treated with a chronic and intensive IV iloprost regimen." *Rheumatol Int* 37(2): 245-249.
11. Casigliani Rabl, S., et al. (2012). "Long-term cyclic intravenous iloprost in systemic sclerosis: Clinical experience from a single center." *Reumatismo* 64(3): 158-165.
12. Harris, E. S., et al. (2018). "Therapeutic plasma exchange for the treatment of systemic sclerosis: A comprehensive review and analysis." *J Scleroderma Relat Dis* 3(2): 132-152.
13. Fartura Braga Temido, M. H., et al. (2019). "Iloprost infusion through elastomeric pump in the treatment of Raynaud's phenomenon and digital ulcers." *J Scleroderma Relat Disord* 4(1): NP1-NP4.
14. Moinzadeh, P., et al. (2011). "Combination therapy with an endothelin-1 receptor antagonist (bosentan) and a phosphodiesterase v inhibitor (sildenafil) for the management of severe digital ulcerations in systemic sclerosis." *J Am Acad Dermatol* 65(3): e102-e104.
15. Yagi, S., et al. (2011). "Bosentan improves systemic sclerosis-related peripheral circulation insufficiency." *Int J Cardiol* 147(3): 472-475.
16. Jaffe, I. A. (1982). "Nifedipine in digital ulceration in scleroderma." *Arthritis Rheum* 25(10): 1267-1269.
17. Chamaillard, M., et al. (2007). "Bosentan as a rescue therapy in scleroderma refractory digital ulcers." *Arch Dermatol* 143(1): 125-126.
18. Kurgyis, Z., et al. (2011). "Bosentan is effective against digital ulcerations and hyperkeratosis in systemic sclerosis." *Acta Derm Venereol* 91(6): 716-717.
19. Lamprecht, P., et al. (1998). "Efficacy of alprostadil and iloprost in digital necrosis due to secondary Raynaud's phenomenon." *Br J Rheumatol* 37(6): 703-704.
20. Snyder, M. J., et al. (2005). “Resolution of severe digital ulceration during a course of Bosentan therapy.” *Ann Intern Med* 142(9): 802-803.
21. Tillon, J., et al. (2006). “Successful treatment of systemic sclerosis-related digital ulcers and sarcoidosis with endothelin receptor antagonist (bosentan) therapy.” *Br J Dermatol* 154(5): 1000-1002.
22. Lee, W. Y., et al. (2013). “Endothelin receptor antagonists and scleroderma related digital ulcers.” *Acta Reumatol Port* 38(1): 58-59.
23. Baron, M., et al. (1982). “Prostaglandin E1 therapy for digital ulcers in scleroderma.” *Can Med Assoc J* 126(1): 42-45.
24. Bruni, C., et al. (2021). “Prediction and primary prevention of major vascular complications in systemic sclerosis.” *Eur J Int Med* 87: 51-58
25. Abouwda, Y., et al. (2017). “Treatment of digital ulcers in systemic sclerosis: Case series study of thirteen patients and discussion on outcome.” *Rev Assoc Med Bras* 63(5): 422-426.
26. Amanzi, L., et al. (2010). “Digital ulcers in scleroderma: staging, characteristics and sub-setting through observation of 1614 digital lesions.” *Rheumatology (Oxford)* 49(7): 1374-1382.
27. Frantz, C., et al. (2020). "Outcomes of limited cutaneous systemic sclerosis patients: Results on more than 12,000 patients from the EUSTAR database." *Autoimmun Rev* 19(2): 102452.
28. Lee, K. A., et al. (2021). "Effects of Bosentan in the Treatment of Digital Ulcers in Korean Patients With Systemic Sclerosis: A Longitudinal, Multicenter, Uncontrolled Trial." *J Clin Rheumatol* 27(8) e599-e601.
29. Cui, J. et al. (2022). “Efficacy and safety of mesenchymal stem cells in the treatment of systemic sclerosis: a systematic review and meta-analysis.” *Stem Cell Res Ther* 13(1): 118.
30. Keret, S. et al. (2022). “G-CSF treatment for refractory digital ulcers in systemic sclerosis.” *Joint Bone Spine* 89(4): 105348

**Wrong population (n=11)**

1. Winston, E. L., et al. (1983). "Nifedipine as a therapeutic modality for Raynaud's phenomenon." *Arthritis Rheum* 26(10): 1177-1180.
2. Roald, O. K. and E. Seem (1984). "Treatment of Raynaud's phenomenon with ketanserin in patients with connective tissue disorders." *Br Med J* 289(6445): 577-579.
3. Selenko-Gebauer, N., et al. (2006). "Successful treatment of patients with severe secondary Raynaud's phenomenon with the endothelin receptor antagonist bosentan." *Rheumatology (Oxford)* 45 Suppl 3: iii45-48.
4. Funauchi, M., et al. (2009). "Effects of bosentan on the skin lesions: an observational study from a single center in Japan." *Rheumatol Int* 29(7): 769-775.
5. Lee, E. Y., et al. (2014). "Head-to-head comparison of udenafil vs amlodipine in the treatment of secondary Raynaud's phenomenon: a double-blind, randomized, cross-over study." *Rheumatology (Oxford)* 53(4): 658-664.
6. Marasini, B., et al. (2004). "Comparison between iloprost and alprostadil in the treatment of Raynaud's phenomenon." *Scand J Rheumatol* 33(4): 253-256.
7. Mohrland, J. S., et al. (1985). "A multiclinic, placebo-controlled, double-blind study of prostaglandin E1 in Raynaud's syndrome." *Ann Rheum Dis* 44(11): 754-760.
8. Clifford, P. C., et al. (1980). "Treatment of vasospastic disease with prostaglandin E1." *Br Med J* 281(6247): 1031-1034.
9. Duarte, A. C., et al. (2018). "Iloprost infusion through elastomeric pump for the outpatient treatment of severe Raynaud's phenomenon and digital ulcers - a single centre experience." *Acta Reumatol Port* 43(3): 237-238.
10. Seibold, J. R. and A. H. Jageneau (1984). "Treatment of Raynaud's phenomenon with ketanserin, a selective antagonist of the serotonin2 (5-HT2) receptor." *Arthritis Rheum* 27(2): 139-146.
11. O’Reilly, M. J. G., et al. (1979). "Controlled trial of plasma exchange in treatment of Raynaud’s syndrome." *Br Med J* (6171): 1113-1115.

**Wrong intervention (n=4)**

1. Nagarajan, M., (2021). “Targeted high concentration botulinum toxin A injections in patients with Raynaud’s phenomenon: a retrospective single-centre experience.” *Rheumatol Int* 41: 934-949.
2. Guigui, A., (2020). “Treprostinil Hydrogel Iontophoresis in Systemic Sclerosis-Related Digital Skin Ulcers: A Safety Study.” *J Clin Pharmacol* 60(6): 758-767.
3. Ciompi, M. L., et al. (1996). "A placebo-controlled study on urokinase therapy in systemic sclerosis." *Biomed & Pharmacother* 50(8): 363-368.
4. Nakamura, H., et al. (2018). "Autologous haematopoietic stem cell transplantation for Japanese patients with systemic sclerosis: Long-term follow-up on a phase II trial and treatment-related fatal cardiomyopathy." *Mod Rheumatol* 28(5): 879-884.

**Duplicate (n=3)**

1. Seibold, J. R. and A. H. M. Jageneau (1984). "Treatment of Raynaud's phenomenon with ketanserin, a selective antagonist of the serotonin_2_ (5-HT_2_) receptor." *Arthritis Rheum* 27(2): 139-146.
2. Mohrland, J. S., et al. (1985). "A multiclinic, placebo-controlled, double-blind study of prostaglandin E_1_ in Raynaud syndrome." *Ann Rheum Dis* 44(11): 754-760.
3. Martin, M. F. R., et al. (1981). "Prostaglandin E_1_ infusions for vascular insufficiency in progressive systemic sclerosis." *Ann Rheum Dis* 40(4): 350-354.

**Non-English (n=3)**

1. (2006). "A pilot trial of treprostinil for the treatment and prevention of digital ulcers in patients with systemic sclerosis." *Dermatologia Revista Mexicana* 50(3): 117-118.
2. Keller, J., et al. (1984). "[Treatment of Raynaud's phenomenon in scleroderma with a new stable prostacyclin derivative]." *Dtsch Med Wochenschr* 109(38): 1433-1438.
3. Cordioli, E., et al. (1992). "[Effects of long-term iloprost therapy on Raynaud's phenomenon in progressive systemic sclerosis]." *Minerva Med* 83(11): 739-744.

**Supplementary Data S4: Data extraction template**

Study characteristics

- First author
- Year of publication
- Journal
- Study country
- Funding sources
- Declared conflicts of interest
- Study design
- Intervention
- Comparator group
- Inclusion criteria
- Exclusion criteria
- Sample size calculation
- Randomisation
- Length of follow up
- Primary outcome
- Definition of digital ulcer
- Definition of ulcer healing (if appropriate)
- Definition of ulcer flare (if appropriate)
- Secondary outcome(s)
- Blinding
- Loss to follow up / management of missing data

Study population (compared between intervention and comparator groups as appropriate)

- Sample size (n)
- Age
- Sex
- Ethnicity
- Smoking status
- Diffuse / limited subtype
- Overlap syndromes
- Disease duration
- Antibody status
- Co-morbidities
  - Diabetes
  - Peripheral vascular disease
  - Others
- Digital ulcer location
- Ischaemic ulcers (n)
- Traumatic ulcers (n)
- Calcinotic ulcers (n)
- Concurrent therapy
  - Calcium channel antagonists
  - PDE5 inhibitors
  - Endothelin receptor antagonists
  - Anti-platelet agents
  - Anticoagulation
  - Statins
  - Immunosuppression
  - Corticosteroids
- Systemic sclerosis disease manifestations
  - Raynaud’s phenomenon
  - Nailfold capillaroscopy pattern
  - Pulmonary arterial hypertension
  - Interstitial lung disease
  - Scleroderma renal crisis
  - Myocardial disease

Study outcomes (compared between intervention and comparator groups as appropriate)

- Total number of ulcers
  - Baseline & follow up
- Number of new ulcers
- Ulcer measurements
  - Baseline & follow up
- SHAQ scores
- HAQ-DI
- Pain (VAS)
- Cochin Hand Function Scale
- SF-36 PCS & MCS
- Physician global assessment score
- Patient global assessment score
- Digital ulcer complications
- Treatment emergent adverse events

Cost effectiveness analysis

**Supplementary Table S1: Characteristics of included studies of systemic pharmacological treatment of digital ulcers in systemic sclerosis**

| **Author**  **Year** | **Participants(n)**  **Baseline characteristics** | **Intervention** | **Study design** | **Inclusion criteria** | **Definition of digital ulcer** | **Definition of ulcer healing** | **Concomitant therapies** | **Primary outcome** | **Results** |
| --- | --- | --- | --- | --- | --- | --- | --- | --- | --- |
| **Calcium channel antagonists** | | | | | | | | | |
| *Cohort studies* | | | | | | | | | |
| Kahan^1^  1983 | 4  Mean age: 54 years  Mean disease duration: 5⋅5 years | Nifedipine  20mg QID for 6 weeks | Single-blind observational study | Refractory DU | Not stated | Not stated | Nil | Daily frequency of RP and number of digital ulcers | Reduction in mean number of DU 7⋅75 🡪 0⋅5 / patient |
| **PDE-5 inhibitor** | | | | | | | | | |
| *Case series* | | | | | | | | | |
| Brueckner^2^  2010 | 16  dcSSc: 56⋅25%  Mean age: 49⋅94 years  Mean disease duration:8⋅44 years | Sildenafil  Maximally tolerated dose up to 150mg daily  Mean duration of treatment 5⋅2 months | Observational pilot study, no control group | Severe RP or presence of DU | Loss of epidermis and dermis | Not stated | CCB (93⋅75%)  Anti-platelet agent (18⋅75%)  Anti-coagulant (6⋅25%)  IV prostacyclin (81⋅25%)  Immunosuppressant (50%) | Healing of digital ulcer | Total DU count: 49 at baseline 🡪 17 at follow up (p<0⋅01);  Mean DU / patient : 3⋅1 🡪 1⋅1 ;  9 patients developed 12 new DU ;  13/16 had previous or concomitant IV prostacyclin |
| Kumar^3^  2013 | 16  7 patients with DU  dcSSc: 62⋅5%  Mean age: 33⋅5 years  Mean disease duration: 5⋅1 years | Sildenafil  Maximally tolerated dose 75mg-300mg / day  3 months observation | Case series | SSc with PAH | Not stated | Not stated | Not reported | Safety & efficacy of sildenafil | 43% patients complete healing of DU; 57% noted significant improvement |
| Della Rossa^4^  2011 | 15  dcSSc: 20%  Mean age: 61 years  Mean disease duration 14⋅3 years | Sildenafil  Maximally tolerated dose 25-120mg / day  Mean duration of treatment 16⋅2 months | Case series | Failure of DU to respond to standard therapy | Not stated | Not stated | Monthly IV iloprost (33⋅33%) Bosentan (13⋅33%) | Not stated | 53% patients complete healing of DU after mean 7⋅1 months; 20% patients reduction in number and size of DU; 70⋅5% of DU healed |
| **Endothelin receptor antagonists** | | | | | | | | | |
| *Cohort studies* | | | | | | | | | |
| Chang^5^  2021 | 63  49 patients received ERA 11 patients received PDE5i  dcSSc: 54⋅1%  Mean age: 49⋅9 years  Mean disease duration: 7⋅3 years | Comparison of efficacy of PDE5i and ERA therapy | Multi-centre cohort study,  no control group | $\geq$1 active DU | Skin lesion with visually discernible depth and loss of continuity of epithelial coverage that was denuded or covered by scab or necrotic tissue | Completely covered with epithelium | CCB (27⋅0%)  Prostacyclin analogues (4⋅8%) | Mean time to healing: 91⋅3 days (ERA) vs 81⋅3 days (PDE5i), p=0⋅47;  HR for complete healing at 24 weeks in ERA vs PDE5i treatment 0⋅75 (p=0⋅47); HR of new DU with ERA vs PDE5i 0⋅39 (p=0⋅03) | Bosentan: LFT changes (8⋅16%), headache (2⋅04%), drowsiness/fatigue (2⋅04%), flushing (12⋅24%), oedema (12⋅24%), anaemia (4⋅08%)  PDE5i: restrictive heart failure (unclear if related to therapy), flushing (9⋅09%), oedema (9⋅09%), palpitations (9⋅09%) |
| Hamaguchi^6^  2017 | 28  dcSSc: 68%  Mean age: 57⋅8 years  Mean disease duration 8⋅5 years | Bosentan  62⋅5mg BD for 4 weeks then increased to 125mg BD from week 5 onwards.  52 week study | Multicentre, open label observational study, no control group | RP and at least one active DU at time of consent or history of DU within 5 years | Ulcers with keratolysis involving distal to PIP of fingers or IP joint of thumb, calcium extrusions excluded | Complete healing: formation of epithelium visually confirmed by investigator | Vasodilators | Safety of bosentan | Mean number of DU at baseline 1⋅2 🡪 0⋅6 at week 16; new DU occurred in 25% patients over 16 weeks; complete healing in 45⋅45% patients with DU at baseline |
| Kuhn^7^  2010 | 10  dcSSc:  Mean age: 58⋅9 years  Mean disease duration: 10⋅94 years | Bosentan  62⋅5mg BD for 4 weeks increased to 125mg BD for 20 weeks; 24 week observational period | Prospective, open-label observational study, no control group | Current areas of skin fibrosis and total mRSS >14 | Loss of surface epithelium at or distal to PIP joint, did not include fissures or cracks in skin or areas calcium extrusion | Total re-epithelialisation | Corticosteroids (50%)  Immunosuppressant (10%) | Change in mRSS; number of digital ulcers secondary end point | Change in percentage of patients with healed ulcers: Week 0 - 42% 🡪 Week 24 - 88% (p=0⋅002) |
| *Case series* | | | | | | | | | |
| Mouthon^8^  2017 | 120  dcSSc: 42⋅6%  Mean age: 54⋅2 years  Mean disease duration: 8⋅0 years | Bosentan  Mean daily dose 195mg daily  12 month follow up | Multi-centre, prospective observational study | SSc with at least 1 DU and treatment with bosentan | DU on pulpar surface of fingers, distal to PIP and not facing calcinosis or bone profile. | Not stated | CCB (60⋅9%)  IV iloprost (18⋅4%) | No active digital ulcers and no new digital ulcers over period of follow up | 38⋅3% patients had new DU over 12 months; incidence of new DU 0⋅6/patient year; proportion of patients with $\geq$1 active DU 60⋅8% 🡪 22⋅5% at 1 year (p<0⋅001); mean number of DU per patient 1⋅4 🡪 0⋅6 (p<0⋅001) |
| Kucuksahin^9^  2016 | 30  dcSSc: 66⋅7%  Mean age: 49⋅6 years; Mean disease duration: 9⋅3 years | Bosentan  Dosage  62⋅5mg BD for one month then increased to 125mg BD for up to 24 months; mean follow up 14 months | Observational study, no control group | DU refractory to treatment with CCB or ARB | Marginated lesion and ischaemic gangrene ulcers | Not stated | IV iloprost (40%) for treatment of new digital ulcer or critical digital ischaemia | Number of ulcers, number of new DU | 8 (26⋅7%) patients developed new digital ulcers on treatment |
| Tsifetaki^10^  2009 | 26  dcSSc: 62⋅5%  Mean age: 60 years  Mean disease duration: 12⋅5 years | Bosentan  62⋅5mg BD for first month then increased to 125mg BD for 36 months | Prospective cohort study, no control group | DU refractory to CCB, angiotensin II inhibitors or sildenafil | Not stated | Not stated | Corticosteroids (100%)  Immunosuppressants (88.46%) | Number of healed digital ulcers and number of new skin ulcers | Mean baseline DU: 5⋅8 🡪 3⋅8 at 36 months (p<0.001)  Healing of DU in 65% patients after median 25 weeks  19% patients developed new ulcers |
| Chung^11^  2014 | 20  Mean age: 49⋅3 years | Ambrisentan  Up to 10mg daily as tolerated for 24 weeks | Open label, no control group | $\geq$1 active DU with onset within 12 weeks of screening | DU on or distal to PIP joint | Not stated | Stable dose vasodilator (except PDE-5 inhibitor, other ERA or prostacyclins) | Total number of digital ulcers, mean diameter of ulcer | Number of ulcers per patient 3⋅1 🡪 1⋅3 (p=0⋅04); reduction mean maximum diameter 3⋅3 🡪 1⋅6mm (p<0⋅001); 3⋅2 new ulcers per patient over 24 weeks |
| de la Pena-Lefebvre^12^  2008 | 15  dcSSc: 33⋅3%  Mean age: 48 years  Mean disease duration: 14⋅5 years | Bosentan  62⋅5mg BD for 4 weeks then increased to 125mg BD for up to 36 months | Case series | DU refractory to conventional treatment | Not stated | Not stated | CCB (53⋅33%)  ARB (46⋅67%)  IV prostaglandin (13⋅33%)  Inhaled iloprost (6⋅67%) | Not stated | Decreased number of ulcers at 6 months (-2⋅8, p=NS), 12 months (-4⋅6, p<0⋅05), 24 months (-4⋅9, p<0⋅05);  Decreased number of healed ulcers at 6 months (-2⋅6, p<0⋅05), 12 months (-2⋅4, p=NS), 24 months (-3⋅7, p=NS) |
| Nagai^13^  2012 | 6  dcSSc: 83⋅33%  Mean age: 41⋅17 years  Mean disease duration: 9⋅33 years | Bosentan  62⋅5mg – 125mg  Follow up period 2 months to 4⋅5 years  Median follow up:11⋅5 months | Case series | Received bosentan for management of DU | Not stated | Not stated | Lipo-prostaglandin E_1_ (50%) hyperbaric oxygen therapy (33%) | Not stated | No new digital ulcer developed on therapy |
| Parisi^14^  2013 | 6  dcSSc: 33⋅33%  Mean age: 53⋅7 years  Mean disease duration: 7⋅1 years | Ambrisentan  5mg / day; follow up for 6 months | Case series | DU refractory to bosentan; onset of DU between 6-9 months | Not stated | Not stated | ACEI (100%)  IV Prostacyclin analogues  (100%) | Digital ulcer healing | 4/6 patients had complete healing of ulcers  Mean number of ulcers/patient 2⋅67 🡪 0⋅33, p<0⋅03; no new digital ulcers observed |
| **Prostacyclin analogues** | | | | | | | | | |
| *Cohort studies* | | | | | | | | | |
| Shah^15^  2016 | 51  dcSSc: 15⋅7%  Mean age: 53⋅8 years  Mean disease duration: 12⋅3 years | Treprostinil (oral)  Follow up of study participants up to 12 months after discontinuation of treatment (withdrawal trial) | Observational cohort study | Active DU; follow up data available following completion of study | An area with visually discernible depth and loss of continuity of epithelial coverage, distal to PIP, volar to equator of finger, not localised to IP creases and not related to calcinosis | Not stated | CCB (60⋅8%) PDE5i (21⋅6%) | Changes in DU burden | Mean active DU: baseline 0⋅47 🡪 up to 6 months: 2⋅1 (p=0⋅002);  In 30 patients with >6 months follow up: mean active DU baseline 0⋅4 🡪 up to 12 months: 1⋅45 (p=0⋅013) |
| Shenavandeh^16^  2022 | 26  dcSSc: 23%  Median age: 45⋅5 years | IV alprostadil (60$\mu$g for 3 days or 250$\mu$g over 48 hrs) or iloprost (20$\mu$g for 3 days) compared to botulinium toxin-A (20 units each finger) | Comparative study, open label | SSc with active DU | Visible de-epithelialised base on volar or lateral side of distal finger | Total re-epithelialisation of an ulcer | Prednisolone (73%)  CCB (100%)  PDE5i (38%) | Not stated | 90⋅5% patients had healing of DU after prostaglandin analogue  91⋅5% patients had healing of DU after botulinium toxin-A |
| *Case series* | | | | | | | | | |
| Bettoni^17^  2002 | 30  Median age: 55 years  Median disease duration: 10 years | Iloprost (IV)  5 consecutive days then 1 day every 3 weeks; median duration of follow up 36⋅5 months | Cohort study,  No control group | Severe RP, ischaemic ulceration, pulmonary hypertension (n=1) | Ischaemic lesions not further defined | Not stated | CCB (57%)  ACEI (10%)  ARB (3⋅33%)  Aspirin (70%)  Corticosteroids (37%)  Immunosuppressant (20%) | Number of DU, RP severity, mRSS, HAQ | Baseline: 22/30 patients had DU; 90% had complete healing |
| Rademaker^18^  1987 | 13  Mean age: 49 years  Mean disease duration: 16 years | Iloprost (IV)  2ng/kg/min for 8 hours on 3 consecutive days | Case series | SSc and severe RP | Digital lesions – DU or fissures | Not stated | Nil | Peripheral blood flow assessment | Improved PVR and increased digital blood flow. Reduction in cutaneous lesions from 26 🡪 14 (2 weeks after infusion) 🡪 7 (10 weeks) |
| Del Papa^19^  2020 | 6  dcSSc: 100%  Mean age: 42⋅83 years  Mean disease duration: 8 years | Selexipag  2400-3000mcg / day  3-6 months | Case series | DU uncontrolled with CCB, ERA, PDE5 or IV prostanoid & inadequate IV access | Not stated | Not stated | Aspirin (66⋅67%)  ERA (83⋅33%)  PDE5i (83⋅33%)  Immunosuppressants (66⋅67%) | Not stated | 100% resolution of DU and associated pain, 88⋅3% improvement of RP burden |
| Zachariae^20^  1996 | 6 | Iloprost (IV)  0⋅5-2⋅0ng/kg/min (maximum tolerated dose) for 6 hr from 8-13 days | Observational, case series, no control | SSc | Ischaemic ulcer | Healing of all ulcers present at baseline | Prior therapy with nifedipine and immunosuppressants permitted. | Not stated | 66⋅67% patients had DU healing |
| **Antiplatelet agents** | | | | | | | | | |
| *Case series* | | | | | | | | | |
| Ntelis^21^  2016 | 13  dcSSc: 38⋅46%  Mean age: 59⋅5 years  Mean disease duration: 10⋅9 years | Clopidogrel  75mg daily for 12 months | Proof of concept study | Patients with SSc | Not stated | Not stated | ERA (31%)  Corticosteroids (23%)  Immunosuppressant (62%) | ADP-dependent platelet activation suppression with treatment, circulating levels of serotonin | 3 patients developed new onset DU on therapy |
| **Statins** | | | | | | | | | |
| *Cohort studies* | | | | | | | | | |
| Kuwana^22^  2009 | 8  3 patients with DU at enrolment  dcSSc: 50%  Mean age: 61⋅7 years  Mean disease duration 12⋅75 years | Atorvastatin  10mg / day;  24 months | Open label observational study | SSc and assessment of total serum cholesterol level | Not stated | Not stated | Beraprost (oral) only (50%)  Beraprost (oral) + CCB (50%)  Corticosteroids (12⋅5%) | Effects on RP | Mean number of DU prior to study entry 4⋅7 / year 🡪 3⋅3 / year at 12 months 🡪 2⋅7 / year at 24 months |
| **Immunosuppression and other therapies** | | | | | | | | | |
| *Cohort studies* | | | | | | | | | |
| Hou^23^  2022 | 10  4 with DU  Mean age: 40  Mean disease duration: 19.5 months | Baricitinib 2mg or 4mg daily  24 weeks | Open label, uncontrolled clinical trial | Definite SSc | Epithelialisation loss on distal surface of finger causes by skin rupture or ischaemia. Not located over calcification or joint extensor. | Not stated | Prednisolone (30%)  Nifedipine (30%) | mRSS: -11·67, p<0·01 over 24 weeks | 3/4 (75%) patients with DU complete resolution at 24 weeks |
| *Case series* | | | | | | | | | |
| Rosato^24^  2009 | 50  dcSSc: 70%  Mean age: 51 years  Mean disease duration: 9⋅5 years | N-acetylcysteine  15mg/kg/hr every 14 days  Median treatment 3 years | Case series | DU resistant to CCB within past 12 months | Loss of surface epithelialization (not including fissures or cracks in skin or areas of calcinosis) at or distal to PIP joint | Not stated | Oral vasodilators and treatment for RP | Number of DU during pre-treatment period and treatment period | Mean DU/patient/year: 4⋅5 🡪 0⋅81 (p<0⋅01) |
| Sambo^25^  2001 | 22  17 patients with DU at entry  dcSSc: 13⋅63%  Mean age: 49⋅63 years  Mean disease duration: 12⋅18 years | N-acetylcysteine  Loading dose (150mg/kg) followed by 15mg/kg/hr infusion for 5 days  Follow up over 61 days | Observational cohort study, no control group | $\geq$7 RP attack/week or $\geq$1 cutaneous ischaemic lesion | Marginated lesion that results from destruction of epidermis and dermis and considered ischaemic in origin | Not stated | Nil | Mean weekly number of RP attacks, number of DU ulcers present | DU / patient 2⋅94 🡪 1⋅53 (p=0⋅001) |
| Dau^26^  1981 | 15  8 patients had DU at baseline  Mean age: 40⋅6 years  Mean disease duration: 4⋅14 years | Plasmapheresis  Weekly plasma exchange of 5-6% of body weight for up to 10 exchanges with ongoing 1-4 weekly plasma exchange according to patient symptoms and IVIg (range of follow up 1 month-24 months) | Case series | SSc requiring treatment | Not stated | Not stated | Corticosteroids (100%)  Other immunosuppressants (100%) | Not stated | Healing of DU in 87⋅5% of patients. Improvement in DU pain in patient with persistent DU. |
| Klimiuk^27^  1989 | 11  6 with DU  Mean age: 48⋅8 years | Ketanserin – IV (bolus + 72hr 2mg/hr infusion) followed by 6 months PO therapy (40mg TDS) | Observational,  pharmacokinetics study | Patients with CREST and acute on chronic digital ischaemia | Not stated | Not stated | Corticosteroids (9⋅09%) | Post-dose ketanserin blood concentration | Ischaemic ulcers healed in 6/6 patients |
| Mascaro^28^  1987 | 10  3 patients with DU at baseline  dcSSc: 100%  Mean age: 55 years | Plasmapheresis  Twice weekly for 4-6 weeks 2-3 times per year  Range of follow up 1-42 months | Case series | SSc requiring treatment | Finger ulcerations, not further specified | Not stated | ACEI (10%)  Corticosteroids (100%)  Cyclophosphamide (20%)  Griseofulvin (50%) | 66⋅67% patients had resolution of DU with treatment | 66⋅67% patients had resolution of DU with treatment |
| **Combination therapy** | | | | | | | | | |
| *Cohort studies* | | | | | | | | | |
| Trombetta^29^  2016 | 15  dcSSc: 53⋅33%  Mean age: 66 years  Mean disease duration: 14 years | IV iloprost  Iloprost: 50 microgram/day for 5 continuous days every 3 months with add on bosentan  125mg/day for first month then 125mg BD (if developed DU or PAH) | Open label observational study | Severe RP | Not stated | Not stated | Pre-existing treatment with anti-hypertensives, acetyl-salicylic acid and immunosuppressants continued. | Reduction in incidence of new DU (66⋅67% 🡪 13⋅33%, p=0⋅002) | Reduction in incidence of new DU (66⋅67% 🡪 13⋅33%, p=0⋅002) |

*Study included one patient with mixed connective tissue disease

*Abbreviations:* ACEI: angiotensin converting enzyme inhibitor; ADP: adenosine diphosphate; ARB: angiotensin receptor antagonist; BD: twice daily; CCB: calcium channel antagonist; CREST: calcinosis, Raynaud’s phenomenon, esophageal dysmotility, sclerodactyly, telangiectasia syndrome; dcSSc: diffuse cutaneous systemic sclerosis; DU: digital ulcer; ERA: endothelin receptor antagonist; HR: hazard ratio; IP: interphalangeal; IV: intravenous; IVIg: intravenous immunoglobulin; LFT: liver function tests; mRSS: modified Rodnan Skin Score; NS: not significant; NSAID: non-steroidal anti-inflammatory drug; PAH: pulmonary arterial hypertension; PDE5i: phosphodiesterase type 5 inhibitor; PIP: proximal interphalangeal joint; PVR: peripheral vascular resistance; QID: four times daily; RCT: randomized controlled trial; RP: Raynaud’s phenomenon; SSc: systemic sclerosis; TDS: three times daily

**References**

1. Kahan A, Amor B, Menkes CJ, Weber S. Nifedipine in digital ulceration in scleroderma. *Arthritis Rheum* 1983; **26**(6): 809.
2. Brueckner CS, Becker MO, Kroencke T, et al. Effect of sildenafil on digital ulcers in systemic sclerosis: Analysis from a single centre pilot study. *Ann Rheum Dis* 2010; **69**(8): 1475-8.
3. Kumar U, Sankalp G, Sreenivas V, Kaur S, Misra D. Prospective, open-label, uncontrolled pilot study to study safety and efficacy of sildenafil in systemic sclerosis-related pulmonary artery hypertension and cutaneous vascular complications. *Rheumatol Int* 2013; **33**(4): 1047-52.
4. Della Rossa A, Doveri M, D'Ascanio A, et al. Oral sildenafil in skin ulcers secondary to systemic sclerosis. *Scand J Rheumatol* 2011; **40**(4): 323-5.
5. Chang SH, Jun JB, Lee YJ, et al. A clinical comparison of an endothelin receptor antagonist and phosphodiesterase-5 inhibitors for treating digital ulcers of systemic sclerosis. *Rheumatology (Oxford)* 2021; **60**(12): 5814-19
6. Hamaguchi Y, Sumida T, Kawaguchi Y, et al. Safety and tolerability of bosentan for digital ulcers in Japanese patients with systemic sclerosis: Prospective, multicenter, open-label study. *J Dermatol* 2017; **44**(1): 13-7.
7. Kuhn A, Haust M, Ruland V, et al. Effect of bosentan on skin fibrosis in patients with systemic sclerosis: A prospective, open-label, non-comparative trial. *Rheumatology (Oxford)* 2010; **49**(7): 1336-45.
8. Mouthon L, Carpentier PH, Lok C, et al. Controlling the digital ulcerative disease in systemic sclerosis is associated with improved hand function. *Semin Arthritis Rheum* 2017; **46**(6): 759-66.
9. Küçükşahin O, Yildizgören MT, Gerede DM, Maraş Y, Erten Ş. Bosentan For Digital Ulcers in Patients With Systemic Sclerosis: Single Center Experience. *Arch Rheumatol* 2016; **31**(3): 229-33.
10. Tsifetaki N, Botzoris V, Alamanos Y, Argyriou E, Zioga A, Drosos AA. Bosentan for digital ulcers in patients with systemic sclerosis: a prospective 3-year followup study. *J Rheumatol* 2009; **36**(7): 1550-2.
11. Chung L, Ball K, Yaqub A, Lingala B, Fiorentino D. Effect of the endothelin type A-selective endothelin receptor antagonist ambrisentan on digital ulcers in patients with systemic sclerosis: results of a prospective pilot study. *J Am Acad Dermatol* 2014; **71**(2): 400-1.
12. de la Pena-Lefebvre PG, Rubio SR, Exposito MV, et al. Long-term experience of bosentan for treating ulcers and healed ulcers in systemic sclerosis patients. *Rheumatology (Oxford)* 2008; **47**(4): 464-6.
13. Nagai Y, Hasegawa M, Hattori T, Okada E, Tago O, Ishikawa O. Bosentan for digital ulcers in patients with systemic sclerosis. *J Dermatol* 2012; **39**(1): 48-51.
14. Parisi S, Peroni CL, Laganà A, et al. Efficacy of ambrisentan in the treatment of digital ulcers in patients with systemic sclerosis: a preliminary study. *Rheumatology (Oxford)* 2013; **52**(6): 1142-4.
15. Shah AA, Schiopu E, Chatterjee S, et al. The Recurrence of Digital Ulcers in Patients with Systemic Sclerosis after Discontinuation of Oral Treprostinil. *J Rheumatol* 2016; **43**(9): 1665-71.
16. Shenavandeh S, Sepaskha M, Dehghani S, Nazarinia M. A 4-week comparison of capillaroscopy changes, healing effect, and cost-effectiveness of botulinium toxin-A vs prostaglandin analog infusion in refractory digital ulcers in systemic sclerosis. *Clin Rheumatol* 2022; **41**(1): 95-104.
17. Bettoni L, Geri A, Airo P, et al. Systemic sclerosis therapy with iloprost: A prospective observational study of 30 patients treated for a median of 3 years. *Clin Rheumatol* 2002; **21**(3): 244-50.
18. Rademaker M, Thomas RH, Provost G, Beacham JA, Cooke ED, Kirby JD. Prolonged increase in digital blood flow following iloprost infusion in patients with systemic sclerosis. *Postgrad Med J* 1987; **63**(742): 617-20.
19. Del Papa N, Vitali C, Bilia S, et al. Selexipag may be effective in inducing digital ulcer healing in patients with systemic sclerosis. *Clin Exp Rheumatol* 2020; **38**(Suppl 125): S181-S2.
20. Zachariae H, Halkier-Sorensen L, Bjerring P, Heickendorff L. Treatment of ischaemic digital ulcers and prevention of gangrene with intravenous iloprost in systemic sclerosis. *Acta Derm Venereol* 1996; **76**(3): 236-8.
21. Ntelis K, Gkizas V, Filippopoulou A, et al. Clopidogrel treatment may associate with worsening of endothelial function and development of new digital ulcers in patients with systemic sclerosis: results from an open label, proof of concept study. *BMC Musculoskelet Disord* 2016; **17**: 213.
22. Kuwana M, Okazaki Y, Kaburaki J. Long-term beneficial effects of statins on vascular manifestations in patients with systemic sclerosis. *Mod Rheumatol* 2009; **19**(5): 530-5.
23. Hou Z, Su X, Han G, et al. JAK1/2 inhibitor baricitinib improves skin fibrosis and digital ulcers in systemic sclerosis. *Fron Med* 2022; **9**: 859330.
24. Rosato E, Borghese F, Pisarri S, Salsano F. The treatment with N-acetylcysteine of Raynaud's phenomenon and ischemic ulcers therapy in sclerodermic patients: a prospective observational study of 50 patients. *Clin Rheumatol* 2009; **28**(12): 1379-84.
25. Sambo P, Amico D, Giacomelli R, et al. Intravenous N-acetylcysteine for treatment of Raynaud's phenomenon secondary to systemic sclerosis: a pilot study. *J Rheumatol* 2001; **28**(10): 2257-62.
26. Dau PC, Kahaleh MB, Sagebiel RW. Plasmapheresis and immunosuppressive drug therapy in scleroderma. *Arthritis Rheum* 1981; **24**(9): 1128-36.
27. Klimiuk PS, Kay EA, Mitchell WS, et al. Ketanserin: an effective treatment regimen for digital ischaemia in systemic sclerosis. *Scand J Rheumatol* 1989; **18**(2): 107-11.
28. Mascaro G, Cadario G, Bordin G, et al. Plasma exchange in the treatment of nonadvanced stages of progressive systemic sclerosis. *J Clin Apher* 1987; **3**(4): 219-25.
29. Trombetta AC, Pizzorni C, Ruaro B, et al. Effects of Longterm Treatment with Bosentan and Iloprost on Nailfold Absolute Capillary Number, Fingertip Blood Perfusion, and Clinical Status in Systemic Sclerosis. *J Rheumatol* 2016; **43**(11): 2033-41.

**Supplementary Table S2: Risk of bias assessment of included studies**

*Risk of Bias Assessment for Included Randomized Controlled Trials (Cochrane RoB2 Tool)*

**
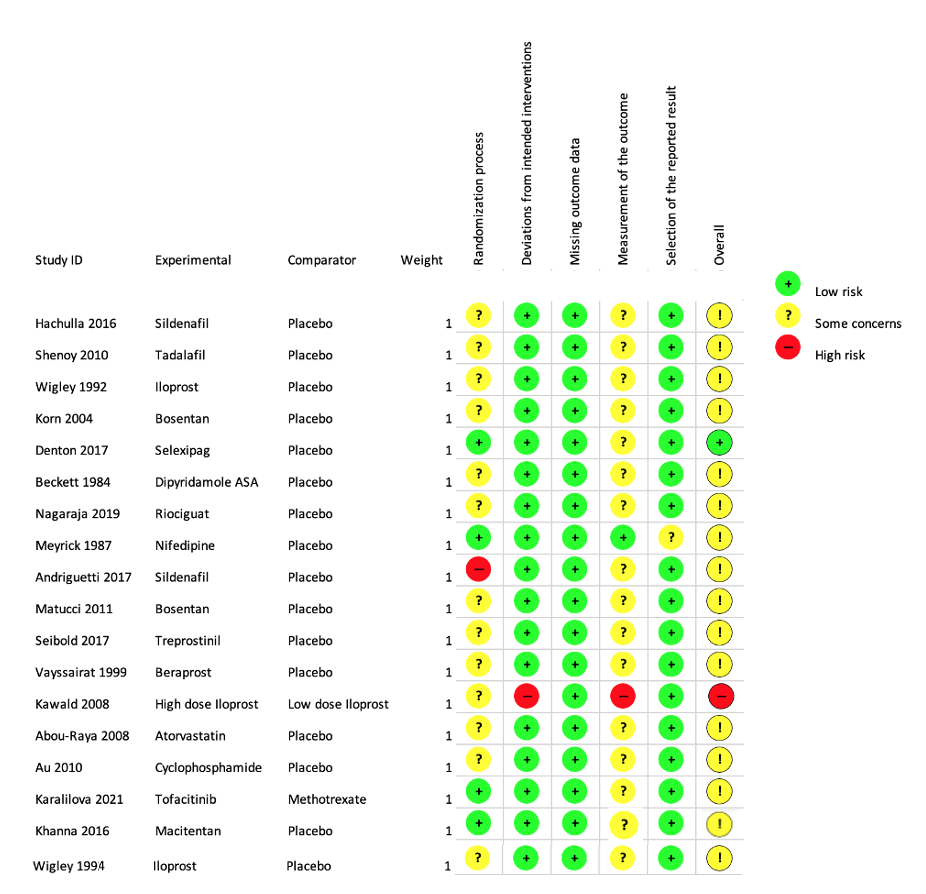
**

*Risk of Bias Assessment of Included Observational Cohort Studies (ROBINS-I)*

| **Study / First author** | **Year** | **Overall Assessment** |
| --- | --- | --- |
| Kahan | 1983 | **Moderate** |
| Kuhn | 2010 | **Moderate** |
| Trombetta | 2016 | **Low** |
| Shah | 2016 | **Moderate** |
| Hamaguchi | 2017 | **Moderate** |
| Chang | 2021 | **Moderate** |
| Shenavandeh | 2021 | **High** |
| Hou | 2022 | **Moderate** |
